# Telomere Dysfunction and Proteostasis Decline Define Distinct Pathways of Cellular Senescence in the Human Respiratory Tract

**DOI:** 10.1111/acel.70512

**Published:** 2026-04-20

**Authors:** Céline Coquette, Kamar Bouchoucha, Manon Mahieu, Stijn E. Verleden, Axelle Loriot, Kevin Norris, Duncan M. Baird, Amélie Derumier, Delphine Hoton, Laurens De Sadeleer, Arno Vanstapel, Bart M. Vanaudenaerde, Maëlle de Ville de Goyet, Bénédicte Brichard, Antoine Froidure, Wim Wuyts, Jan Van Slambrouck, Laurens J. Ceulemans, William Trigaux, Caroline Huart, Anabelle Decottignies

**Affiliations:** ^1^ Genetic and Epigenetic Alterations of Genomes, Telomere Research Group, de Duve Institute, UCLouvain Brussels Belgium; ^2^ Laboratory of Respiratory Diseases and Thoracic Surgery (BREATHE); Department of CHROMETA KU Leuven Leuven Belgium; ^3^ Computational Biology and Bioinformatics, de Duve Institute, UCLouvain Brussels Belgium; ^4^ Division of Cancer and Genetics School of Medicine, Cardiff University Cardiff UK; ^5^ Department of Anatomopathology Cliniques Universitaires Saint‐Luc Brussels Belgium; ^6^ Department of Pathology University Hospitals Leuven Leuven Belgium; ^7^ Department of Pediatric Hematology and Oncology Institut Roi Albert II, Cliniques Universitaires Saint‐Luc, UCLouvain Brussels Belgium; ^8^ Institut de Recherche Expérimentale et Clinique, UCLouvain Brussels Belgium; ^9^ Pulmonology Department Cliniques Universitaires Saint‐Luc Brussels Belgium; ^10^ Department of Respiratory Diseases, Unit for Interstitial Lung Diseases University Hospitals Leuven Leuven Belgium; ^11^ Department of Otorhinolaryngology, Head and Neck Surgery Cliniques Universitaires Saint‐Luc Brussels Belgium; ^12^ OlfactIoNS Group, Institute of Neuroscience (IoNS), UCLouvain Brussels Belgium

**Keywords:** aging, cellular senescence, IPF, lung, nasal epithelium, proteostasis decline, telomere

## Abstract

As the global population ages, cellular senescence contributes increasingly to the burden of age‐related diseases. Hallmarks of this process include telomere shortening and loss of proteostasis, frequently linked to DNA damage‐associated transcriptional stress. Although telomere dysfunction‐induced foci (TIF) have been well documented in lungs from patients with idiopathic pulmonary fibrosis (IPF), their occurrence and role during physiological lung aging remain unclear. Analysis of senescence markers in lung tissue from organ donors aged 16–88 years showed a linear decline in telomere length with age; however, TIF frequency increased significantly in the airway epithelium only in individuals older than 75 years. Similarly, senescence markers such as p16 tended to rise with age but did not reach the levels observed in IPF lungs. To better delineate the early events driving senescence in the human respiratory epithelium and to expand the cohort size, we collected nasal epithelial cells by brushing from 213 healthy volunteers aged 2–97 years. As in the aging lung, telomere shortening was evident, yet TIF were rare and detected almost exclusively in individuals over 80 years of age. In contrast, indicators of impaired proteostasis, including increased senescence‐associated β‐galactosidase activity and lysosomal content, were apparent from the age of 40 in nasal epithelial cells and correlated with olfactory decline. Together, these findings suggest that telomere dysfunction is unlikely to be the primary driver of cellular senescence in the human respiratory tract, where proteotoxic stress may instead play a more prominent role.

## Graphical abstract

Telomere shortening occurs with age in the respiratory epithelium, but telomere dysfunction‐induced foci (TIF) accumulate only late in life. In contrast, proteostasis decline emerges earlier and correlates with functional impairment, suggesting it may be a primary driver of cellular senescence during physiological aging.

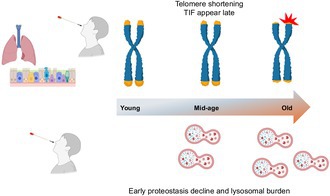

## 1 Introduction

Aging is the primary global risk factor for disease and death, marked by a progressive loss of physiological function. In the human respiratory tract, aging increases susceptibility to lung diseases, impairs olfactory function, and heightens vulnerability to viral infections. The nasal epithelium serves as a crucial first line of defense against inhaled pathogens, allergens, and environmental irritants, and plays a central role in the pathogenesis of both acute and chronic airway disorders (Balazs et al. 2022). Aging of the upper respiratory tract is strongly associated with reduced olfactory capacity (Doty and Kamath 2014; Doty et al. 1984) and has recently been shown to alter nasal epithelial responses to SARS‐CoV‐2 infection (Woodall et al. 2024). Elucidating the molecular mechanisms that drive respiratory tract aging may pave the way for novel therapeutic strategies aimed at reducing the burden of these conditions.

The discovery of five core hallmarks of aging—genomic instability, telomere shortening, epigenetic changes, loss of proteostasis, and impaired macroautophagy (Lopez‐Otin et al. 2023) – has significantly advanced our understanding of the aging process. However, a central challenge in aging research remains: identifying which of these hallmarks act as primary drivers of cellular aging. This question is further complicated by the fact that cellular senescence is heterogenous, with aging hallmarks displaying organ‐specific and time‐dependent patterns (Cohn et al. 2023; Schaum et al. 2020), suggesting that there is no universal answer.

Although age‐related telomere shortening is well documented in human tissues (Everaerts et al. 2018; Takubo et al. 2010), it is not sufficient on its own to induce telomere dysfunction, as defined by the formation of telomere dysfunction‐induced foci (TIF) and the persistent activation of the DNA damage response, which ultimately results in irreversible cell cycle arrest and the onset of cellular senescence (Fumagalli et al. 2012; Hewitt et al. 2012; Rossiello et al. 2022). TIF have been observed in cells from individuals with telomere biology disorders (TBDs) and during replicative senescence in cultured fibroblasts (Rossiello et al. 2022). Lung cells from patients with pulmonary diseases such as idiopathic pulmonary fibrosis (IPF) also display an increased frequency of TIF (Birch et al. 2015; Schafer et al. 2017), and TIF have occasionally been reported in the context of physiological aging in the human lung (Birch et al. 2015; Schafer et al. 2017). However, whether TIF frequency consistently rises with age and contributes to normal physiological aging remains unclear.

During the past decade, proteotoxic stress has emerged as a potential central factor in cellular senescence induction (Ding et al. 2025; Kaushik and Cuervo 2015; Lopez‐Otin et al. 2013, 2023). With age, misfolded and damaged proteins accumulate, in part due to increased reactive oxygen species (ROS) production. Combined with a decline in lysosomal function, this leads to elevated lysosomal content in aging cells evidenced by the heightened activity of senescence‐associated β‐galactosidase (SA‐β‐Gal) (Dimri et al. 1995). The age‐related decline in macroautophagy efficiency further impairs the clearance of damaged organelles, such as mitochondria, exacerbating ROS levels and cellular damage. In vivo evidence for the role of defective autophagy in aging comes from studies showing that restoring autophagy in aged mouse livers improves mitochondrial function and restores hepatic health (Zhang and Cuervo 2008). Additionally, recent research links proteostasis loss to DNA damage‐induced transcriptional stress, resulting in widespread gene expression downregulation in aging cells (Gyenis et al. 2023; Stoeger et al. 2022).

In this cross‐sectional study, we evaluated markers of cellular senescence in lung tissues from organ donors across a broad age range, as well as in freshly collected nasal brushing samples. Although telomere shortening was clearly evident in both tissue types, TIF abundance in the airway epithelium only significantly increased in donors aged 75 and older, reaching levels comparable to those found in IPF lungs. In contrast, nasal epithelial cells showed minimal age‐related changes in TIF frequency. However, signs of proteostasis disruption, including elevated SA‐β‐Gal activity and increased lysosomal content, emerged as early as age 40 and were associated with olfactory decline. These findings suggest that although age‐related telomere dysfunction in the lung epithelium may contribute to IPF pathogenesis, it is unlikely to serve as a universal trigger of cellular senescence. Rather, proteotoxic stress appears to play a more prominent role in driving age‐associated tissue dysfunction in the upper respiratory tract.

## 2 Methods

### 2.1 Lung Samples

Unused or declined lungs for transplantation at University Hospital Leuven (Belgium) were obtained from a total of 20 donors (6 females and 14 males, 16–83 years) (Table S1). Collection of donor lungs for research was performed in accordance with Belgian legislation and approved by the local ethics committee (S59648, S61653). Lungs affected by emphysema were excluded. Because standardized in vivo chest CT scans were not available for these donors who lacked pre‐mortem imaging suitable for quantitative emphysema assessment, we excluded emphysema using high‐resolution ex vivo CT of inflated, frozen lung specimens. This approach enables the detection of macroscopic and regional parenchymal destruction characteristic of emphysema. Although microscopic, age‐related alveolar simplification cannot be entirely ruled out, the absence of radiologic evidence of emphysema on ex vivo CT strongly supports the exclusion of emphysematous pathology in the analyzed specimens. Lungs with IPF (*n* = 12, 3 females and 9 males, 53–66 years) were obtained from patients undergoing lung transplantation at University Hospital Leuven (Belgium) (Table S1). IPF was diagnosed through dynamic multidisciplinary discussion according to ATS/ERS guidelines (Raghu et al. 2018). Written informed consent was obtained from all patients, and the study was approved by the local hospital ethical committee (S52174). Lungs were collected as previously described (Verleden et al. 2021). Briefly, lungs were air‐inflated immediately after resection, snap‐frozen in liquid nitrogen fumes, and subsequently sectioned transversally into 2‐cm slices stored at −80°C. For immunohistochemistry, selected cores were formalin‐fixed and paraffin‐embedded (FFPE). Frozen tissue samples were incubated in 10% formalin for 24 h at room temperature before processing with a Leica TP1020 automated tissue processor (Leica Biosystems, Wetzlar, Germany). Samples were then carefully oriented transversally and embedded in paraffin using a Leica Histocore Arcadia system. Additionally, peripheral wedge biopsies were collected from *n* = 13 control donor lungs (7 females and 6 males, 28–88 years) prior to implantation (Table S1) (Van Slambrouck et al. 2025). Biopsies were taken from the lingula of the left upper lobe, in accordance with institutional ethical approval (S67052). The staple line was removed and four small pieces were promptly placed in cryotubes and frozen on dry ice before storage at −80°C.

### 2.2 Nasal Brushing Sample Collection

Healthy volunteers (*n* = 213, 117 females and 96 males, 2–97 years) were recruited from three independent clinical studies, all approved by the local ethics committee (Comité d'Ethique Hospitalo‐Facultaire Saint‐Luc–UCLouvain) under the following references: 2016/21MAR/121, 2021/10NOV/467, and 2022/07FEV/046 (Table S2). Individuals with TBD (*n* = 11, 7 females and 4 males, 18–62 years) carrying pathogenic variants in *TERT*, *RTEL1*, or *TERC* were enrolled from study 2022/07FEV/046 (Table S2). Inclusion criteria for all participants were the absence of any upper respiratory tract infection within the three weeks prior to sampling and no history of chemotherapy or radiotherapy. Written informed consent was obtained from all participants, parents, and/or legal guardians. Assent was obtained from minors when appropriate. The studies were conducted in accordance with institutional ethical guidelines.

Nasal epithelial samples were collected by a trained physician using a standardized brushing technique. A sterile cytology brush was gently inserted into the lower half of each nostril, targeting the middle turbinate and the lower part of the nasal cavity below it. The brush was rotated to obtain epithelial cells, with one brushing performed per nostril. Both brushes were immediately immersed in 1 mL DMEM/F12 medium (11039021, Gibco, Thermo Fisher Scientific) supplemented with 10% FBS (SV30160.03, Cytiva) and 1% Penicillin/Streptomycin solution (PS‐B, Capricorn Scientific). Cells were mechanically dissociated from the brushes and divided into three fractions, with cell numbers adjusted according to the requirements of each downstream application. The first fraction, used for immunofluorescence combined with FISH (IF‐FISH), was applied to slides using a Cytospin centrifuge (7 × *g*, 5 min). The second fraction, intended for SA‐β‐Gal staining, was briefly centrifuged (30 s), the supernatant discarded, and the pellet fixed in 4% formaldehyde (F/1501/PB15, Fisher Scientific) in 1× PBS for 5 min. After a second brief spin (30 s) and supernatant removal, the pellet was resuspended in 1× PBS and applied to slides via Cytospin (113 × *g*, 10 min). The third fraction, reserved for qRT‐PCR analyses, was pelleted (30 s spin) and stored at −80°C.

### 2.3 Telomere Restriction Fragment (TRF) Length Analysis

TRF analyses were performed on genomic DNA extracted from lung tissue samples, as described previously (Episkopou et al. 2019), using 5 μg of RNase‐treated genomic DNA digested with 20 U *HinfI* (R0155S, NEB) and 20 U *RsaI* (R0167L, NEB) and a [γ‐^32^P] ATP (BLU502A250UC, Revvity)‐labeled (*TAACCC*)_4_ probe (Eurogentec). Smart Ladder (MW‐1700‐10, Eurogentec) was run together with the samples, stained with SYBR Safe DNA Gel Stain (S33102, Thermo Fisher), and imaged with a ruler. The blots were exposed to a phosphor screen, scanned on Phosphorimager (GE Healthcare), and quantified using the online available WALTER (Web‐based Analyzer of the Length of Telomeres) software (https://www.ceitec.eu/chromatin‐molecular‐complexes/rg51/tab?tabId=125#WALTER).

### 2.4 Immunofluorescence and Telomeric FISH

The protocol described previously (Episkopou et al. 2019) was adapted for TIF detection on frozen tissue sections. First, 5 μm‐thick sections were briefly incubated in cold acetone for 5 min at room temperature (RT), then washed with 1× PBS before fixation in 4% formaldehyde in 1× PBS for 10 min at RT under gentle agitation. After an additional wash with 1× PBS for 5 min at RT, samples were heated in 10 mM citrate buffer (pH 6.0) for 10 min in a microwave oven (450 W), incubated in permeabilization buffer (20 mM Tris–HCl pH 8.0, 50 mM NaCl, 3 mM MgCl_2_, 300 mM sucrose, 0.5% Triton X‐100) for 1 h at 37°C, and then washed three times for 3 min each in 0.1% Tween‐20 in 1× PBS (PBS‐T). Following these steps, tissue sections were either subjected to immunofluorescence (IF) using anti‐TRF2 (1:2000) to label telomeres and anti‐γ‐H2AX (1:1000) to detect DNA damage, or first processed for telomeric FISH and subsequently stained by IF with γ‐H2AX (1:1000) and pro‐SPC (1:1000) to identify AT2 cells. In the first case, tissue sections were first incubated for 45 min at RT in 400 μL of blocking solution (2% normal goat serum (S26‐M, Sigma‐Aldrich), 0.2% BSA (A7030, Sigma‐Aldrich) and 0.1% Triton X‐100 in 1× PBS) before adding the primary antibodies (Table S3) diluted in the same blocking solution and incubated overnight at 4°C. After three 3‐min washes at 45°C in PBS‐T, samples were incubated for 40 min at 45°C with secondary antibodies (Table S3) diluted 1:400 in blocking solution. Samples were then washed three times for 3 min each in PBS‐T at 45°C, followed by three final washes in 1× PBS at RT. After air drying, samples were mounted with fluorescence mounting medium (S302380‐2, Agilent Technologies) containing 0.1 μg/mL DAPI (D9542, Sigma‐Aldrich). When telomeric FISH was performed, tissue sections were first incubated with RNase A solution (0.1 mg/mL, R4875, Sigma‐Aldrich) in 2× SSC for 1 h at 37°C. After three washes of 3 min each in PBS‐T, slides were dehydrated in successive ethanol baths (70%–80%–90%–100%), 3 min each. Slides were air‐dried before incubation with the FISH hybridization solution (160 nM G‐rich red LNA (5′TYE563/GGGT+TAGGG+T + TAG+GGTTAGGG+T + TAGGG+T + TAGGG+TTA/3′ TYE563, 339414, Qiagen) telomeric probe, 50% deionized formamide (P040.2, Carl Roth), 2× SSC, 1× Blocking reagent (11096176001, Roche)). The first incubation was performed at 83°C for 10 min, then slides were kept at 37°C in the dark for 2 h. Slides were then washed two times for 15 min in 50% formamide, 2× SSC, 20 mM Tris–HCl pH 7.4, and three times for 5 min in 150 mM NaCl, 0.05% Tween‐20, 50 mM Tris–HCl pH 7.4. Finally, slides were dehydrated as above and air‐dried before processing with IF as described above.

For TIF detection in nasal brushing samples, we used the combination of telomeric FISH and IF against γ‐H2AX. Nasal epithelial cells, spread on slides, were first incubated for 5 min in permeabilization buffer, then fixed for 15 min in 4% formaldehyde in 1× PBS, and subsequently re‐incubated in permeabilization buffer for 10 min. IF steps were performed as described above, using antibodies against γ‐H2AX (1:1000) and anti‐mouse Alexa Fluor 647 as secondary antibodies (1:400) (Table S3). After washes, cells were fixed again for 2 min with 4% formaldehyde in 1× PBS, washed three times for 3 min with 1× PBS, and incubated with the RNase A solution for 1 h at 37°C. Slides were washed again three times for 3 min with 2× SSC before incubation in permeabilization buffer for 10 min at RT. After a last fixation for 2 min with 4% formaldehyde in 1× PBS, slides were dehydrated in successive ethanol baths (70%–80%–90%–100%), 2 min each. Slides were air‐dried before incubation with the FISH hybridization solution. The first incubation was performed at 83°C for 3 min, then slides were cooled down to RT for 2 h. Finally, slides were washed two times for 15 min in 50% formamide, 2× SSC, 20 mM Tris–HCl pH 7.4, and then three times for 5 min in 150 mM NaCl, 0.05% Tween‐20, 50 mM Tris–HCl pH 7.4. Slides were dehydrated as above and air‐dried before mounting with fluorescence mounting medium.

For detection of lysosomes and CD68+ cells in nasal brushing samples, spread cells were permeabilized for 30 min at RT in Tris–HCl buffer with 0.5% Triton X‐100, rinsed with 1× PBS, and blocked for 1 h at RT in 1× PBS containing 0.1% Triton X‐100, 0.21% BSA, and 2.1% normal goat serum. Slides were then incubated overnight at 4°C with primary antibodies (Table S3) diluted in blocking solution: LAMP2 (1:200), β‐catenin (1:200), or CD68 (1:200). Samples were incubated three times for 3 min each in PBS‐T at 45°C before incubation for 40 min at RT with secondary antibodies diluted in blocking solution: anti‐mouse Alexa Fluor 647 and anti‐rabbit Alexa Fluor 488 (1:1000). This was followed by another series of three 3‐min washes with PBS‐T at 45°C, and a final series of three 3‐min washes with 1× PBS at RT. Slides were mounted as described above. Images were acquired using a Cell Observer Spinning Disc confocal microscope (Zeiss) with either a 40× or a 100× oil immersion objective. Image processing was performed using ImageJ (imagej.nih.gov).

### 2.5 p16 Immunohistochemistry on Lung Sections

p16 immunochemistry (IHC) was performed on 5 μm‐thick FFPE lung sections according to the manufacturer's instructions using the mouse E6H4 anti‐p16 monoclonal antibody and the VENTANA detection kit (705‐4793, Roche) on a BenchMark ULTRA automated slide stainer. Image acquisition and processing was performed as described above. After IHC, IPF lung sections were stained with Alcian Blue (A3157‐10G, Sigma‐Aldrich) to detect fibroblastic foci.

### 2.6 RNA Extraction and qRT‐PCR

For lung samples, total RNA was extracted from ten 10 μm‐thick cryosections of frozen tissue using TriPure reagent (11667165001, Roche), following the protocol described previously (Episkopou et al. 2019). For nasal brushing samples, total RNA was extracted from cell pellets using the RNeasy Plus kit (74006, Qiagen). Pellets were resuspended in 350 μL RLT buffer containing 1% β‐mercaptoethanol, followed by the addition of 350 μL 70% ethanol. The suspension was transferred to the spin column and centrifuged for 30 s at 8000 × *g* at RT. Bound RNA was washed with 350 μL RW1 buffer and centrifuged for 30 s at 8000 × *g*. On‐column DNase digestion (2.7 U/μL; 79,256, Qiagen) was performed to remove genomic DNA, followed by an additional RW1 wash. Two washes with 500 μL RPE buffer were carried out, each followed by centrifugation (30 s at 8000 × *g*, then 2 min at 8000 × *g*), and a final 1 min centrifugation at maximum speed. RNA was eluted by incubating 25 μL RNase‐free water (RF) on the membrane for 20 min, followed by centrifugation for 30 s at 8000 × *g*. cDNA synthesis and qRT‐PCR analyses were performed as described previously (Episkopou et al. 2019) using primers described in Table S4.

### 2.7 SA‐β‐Gal Activity

For lung samples, the SA‐β‐Gal activity assay was performed at pH 5.6 on 16 μm‐thick sections as described previously (Froidure et al. 2020). Briefly, after fixation with 0.2% formaldehyde for 10 min, sections were rinsed with 1× PBS before overnight incubation at 37°C in staining solution (40 mM citric acid/Na phosphate, pH 5.6, 5 mM [K_4_[Fe(CN)_6_]·3H_2_O (P9387, Sigma‐Aldrich)], 5 mM K_3_[Fe(CN)_6_] (702,587, Sigma‐Aldrich), 150 mM NaCl, 2 mM MgCl_2_, 1 mg/mL X‐gal (B4252, Sigma‐Aldrich)). The next day, slides were scanned using the Mirax Midi Imaging system (Zeiss). Images were analyzed with QuPath (qupath.github.io). For nasal brushing samples, spread cells were first rinsed with 1× PBS before incubation for 18 h at 37°C in the staining solution at pH 6. A positive control was performed at pH 4. After rinsing with 1× PBS and mounting with 90% glycerol in distilled water, images were acquired using an EVOS XL Core microscope (Invitrogen, Thermo Fisher Scientific) in brightfield mode to count blue‐stained cells and in phase‐contrast mode to count total cells, using a 40× objective. Results at pH 6 were excluded if the percentage of blue cells at pH 4 was below 85%.

### 2.8 Single Telomere Length Analysis (STELA)

STELA analysis was performed as described previously (Baird et al. 2003). Briefly, genomic DNA was extracted using QIAmp DNA Micro Kit (56304, Qiagen) and eluted in 35 μL 10 mM Tris–HCl (pH 8) and 1 μL of Telorette2 linker (10 μM) added to the DNA solution. This was diluted to 500 pg/μL. One μL of this diluted DNA/Telorette2 solution were subjected to PCR in a 10 μL reaction containing 0.5 μM 17p telomere‐specific primer (17pseq1rev: 5′‐GAATCCACGGATTGCTTTGTGTAC‐3′), 0.5 μM Teltail primer and 0.5 U of a 10:1 mixture of Taq (Thermo Fisher Scientific) and Pwo polymerase (11644955001, Roche). PCR amplicons were resolved by 0.5% TAE agarose gel electrophoresis and were detected by Southern hybridization with a random‐primed α‐^33^P‐labeled (Hartmann Analytic GmbH) TTAGGG repeat probe together with a probe to detect 1 kb (Stratagene) and 2.5 kb (170–8205, BioRad) molecular weight markers. The blots were exposed to a phosphor screen, scanned on Typhoon FLA 9500 phosphorimager (GE Healthcare) and analyzed using ImageQuant TL (GE Healthcare).

### 2.9 Olfactory Tests

Healthy volunteers (*n* = 72, 42 females and 30 males, 18–97 years) were recruited from two independent clinical studies, all approved by the local ethics committee (Comité d'Ethique Hospitalo‐Facultaire Saint‐Luc–UCLouvain) under the following references: 2016/21MAR/121 and 2022/07FEV/046 (Table S2). Individuals with TBD (*n* = 10, 6 females and 4 males, 18–62 years) carrying pathogenic variants in *TERT*, *RTEL1*, or *TERC* were enrolled from study 2022/07FEV/046 (Table S2). Written informed consent was obtained from all participants, parents, and/or legal guardians. Assent was obtained from minors when appropriate. The studies were conducted in accordance with institutional ethical guidelines.

Olfactory abilities were measured with the Sniffin' Sticks test (200112, MediSense) (Hummel et al. 2017). The test yields three separate scores: odor threshold (T), discrimination (D), and identification (I). The stimuli are presented via felt‐tip pens filled with odor solutions, which are held just below the nostrils and gently moved past them for several seconds. The threshold task involves phenylethanol, serially diluted across 16 concentrations. In each trial, participants are given three pens, only one of which contains the odor. Using a forced‐choice procedure, they attempt to detect the correct pen. A staircase rule was applied, where performance determined whether weaker or stronger concentrations were presented. After seven reversals, the threshold value was computed as the mean of the last four reversals, producing a score between 1 and 16. Discrimination is assessed with 16 sets of three pens, where two pens contain the same odor and the third differs. With eyes closed, participants indicate the odd stimulus. The number of correct responses provides the discrimination score (0–16). For identification, 16 familiar odors—commonly recognized by most healthy individuals—are presented individually. Participants choose the correct label from four options read aloud by the examiner, yielding an identification score between 0 and 16. The overall measure of olfactory performance, the TDI score, is obtained by summing the three subscores, with a maximum of 48 points.

### 2.10 Statistical Analyses

All analyses were performed using the GraphPad Prism software. Graphs were also obtained with GraphPad Prism. The statistical tests that were applied are detailed in the figure legends.

## 3 Results

### 3.1 TIF Increase Occurs Late in the Aging Human Airway Epithelium

**FIGURE 1:**
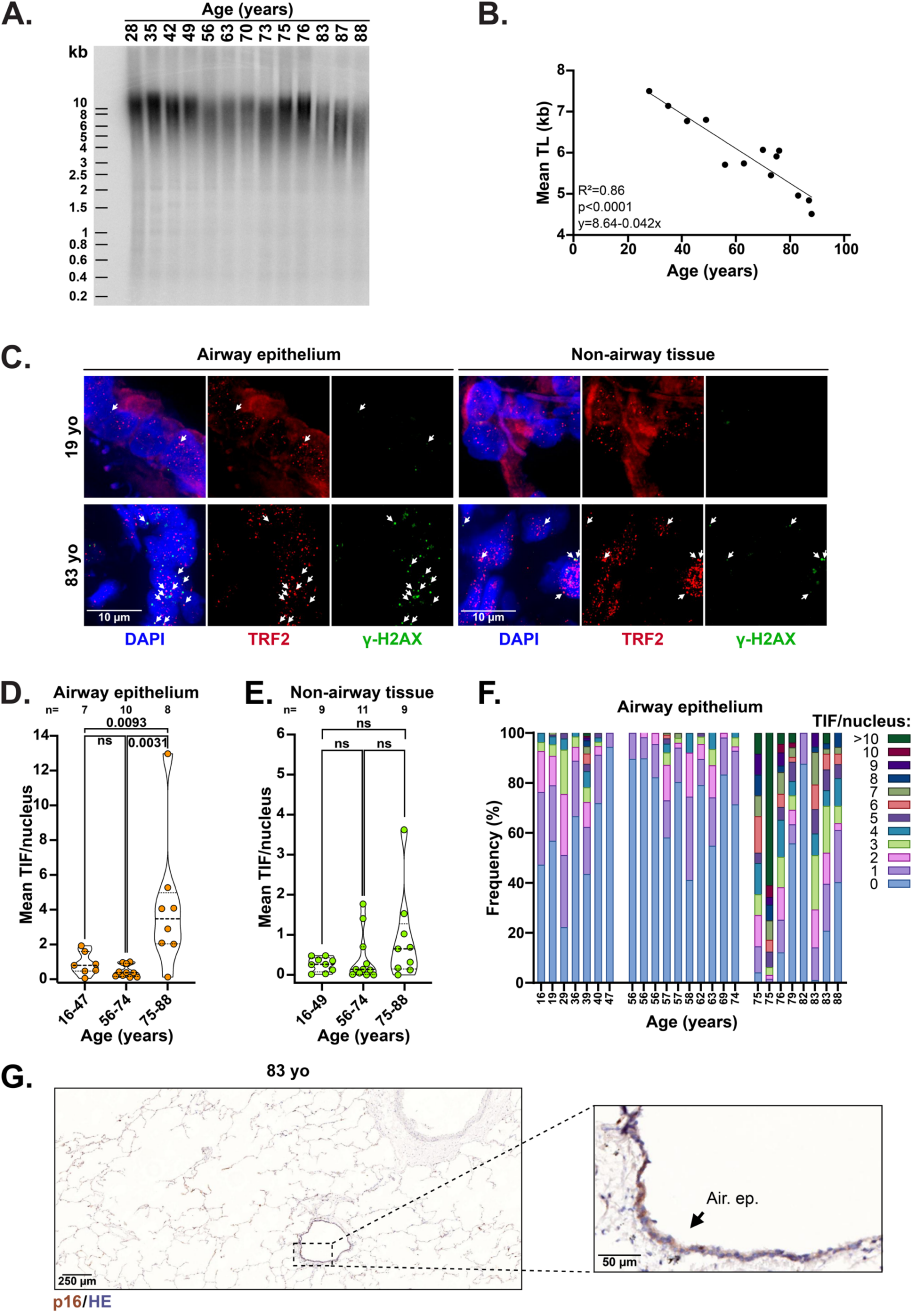
TIF increase occurs late in the aging human airway epithelium. (A) TRF analysis of TL in lung tissues from control donors (Ctrl) aged 28–88 years. Ladder (kb). (B) Linear regression analysis corresponding to panel A. (C) Representative images of TIF analysis in the airway epithelium (left) and non‐airway tissues (right) of control lungs from 19‐ and 83‐year‐old donors. Telomeres are detected with an antibody against TRF2 telomere‐binding protein (red) and DNA damage is detected with an antibody against ɣ‐H2AX (green). DNA is stained with DAPI (blue). Arrows indicate TIF. Scale bar: 10 μm. (D) Mean number of TIF per nucleus in the airway epithelium of donors across the specified age ranges. On average, around 75 nuclei were analyzed per sample. Violin plots showing median, first and third quartile. Mann–Whitney test. (E) As in panel D, but for the non‐airway tissues. On average, around 100 nuclei were analyzed per sample. Mann–Whitney test. (F) Distribution of nuclei by the number of TIF per nucleus in the airway epithelium of donors at the indicated ages. (G) Representative images of p16 staining by immunohistochemistry in lung sample from a 83 years old donor. Air. Ep., airway epithelium; HE, hematoxylin–eosin. Scale bars: 250 and 50 μm.

To monitor TIF formation in the aging human lung, we used tissue samples from organ donors aged 16–88 (Table S1). Consistent with previous findings (Lee et al. 2021; Van Slambrouck et al. 2025), we observed a linear, age‐related decrease in telomere length (TL) (Figure 1A,B). In our lung tissue cohort, the age‐dependent TL attrition was of about 42 ± 5 bp per year, in line with the 20–60 bp range previously proposed for yearly rates of TL reduction in human tissues (Takubo et al. 2010).

TIF are identified by the co‐localization of DNA damage markers with telomeres (d'Adda di Fagagna et al. 2003). Here, TIF were assessed by immunofluorescence‐based co‐detection of telomeres (TRF2) and γ‐H2AX DNA damage marker (Figures 1C and S1A). Although the number of lung tissue samples analyzed was limited, we observed that the average number of TIF per nucleus in airway epithelial cells was significantly higher in tissues from donors aged 75–88 (*n* = 8) compared to those aged 16–47 (*n* = 7), with a median of 3.5 TIF per nucleus in the oldest group (Figure 1D). In contrast, this increase was not significant in the other cell types of the same lung samples (Figure 1C,E). In the oldest airway epithelial tissues, nuclei frequently contained more than one TIF (Figure 1F). In line with the elevated TIF levels in the aging airway epithelium, p16 staining was notably more intense in this region (Figure 1G). These findings were further supported by a trend toward elevated *p16* mRNA expression in lung tissue extracts from aged individuals (Figure S1B,C).

**FIGURE 2:**
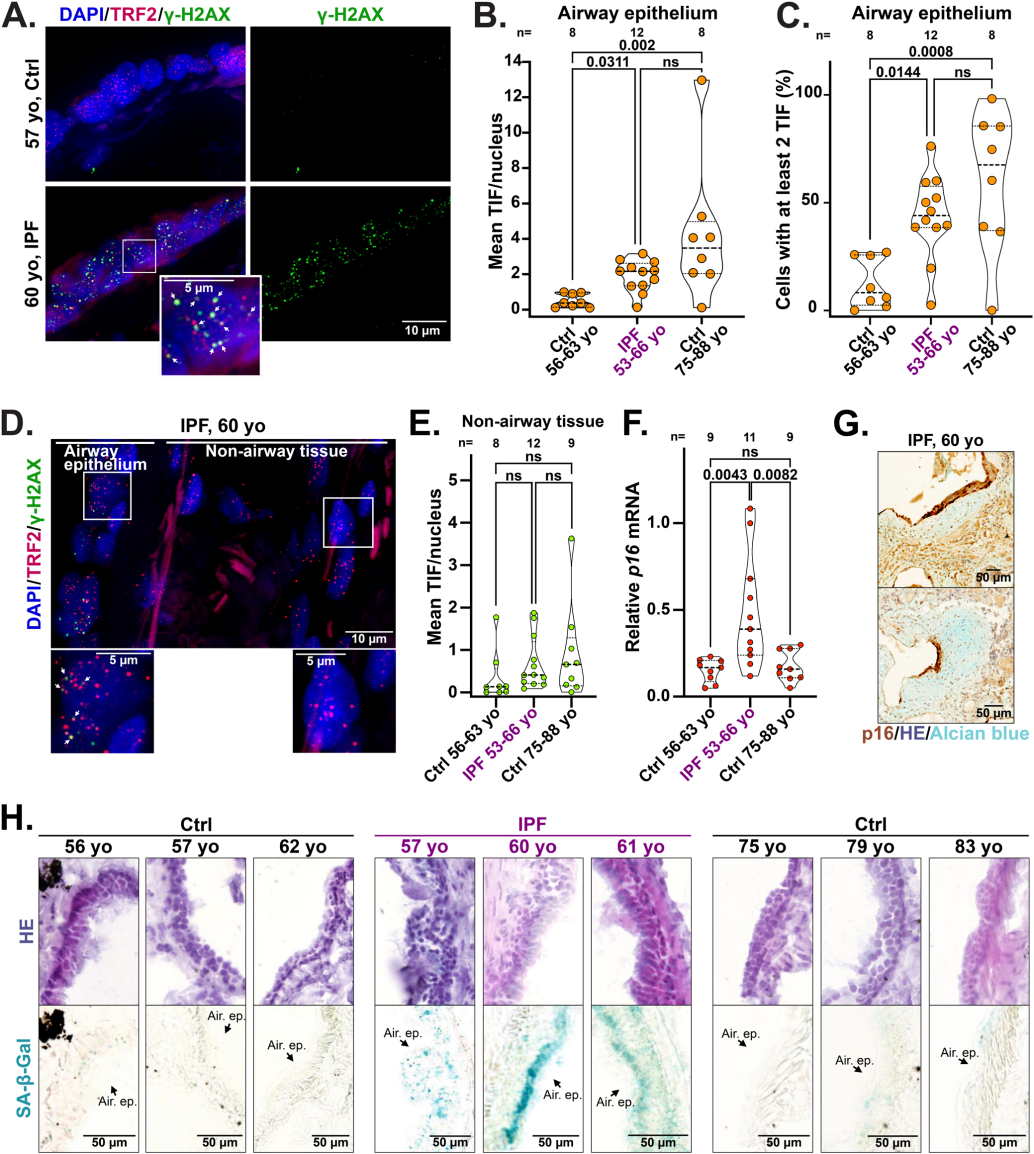
Premature TIF and senescence in IPF airway epithelium. (A) Representative images of TIF analysis in the airway epithelium from a 57‐year‐old control donor (Ctrl) and a 60‐year‐old donor with IPF. Telomeres are detected with an antibody against TRF2 telomere‐binding protein (red) and DNA damage is detected with an antibody against ɣ‐H2AX (green). DNA is stained with DAPI (blue). Arrows indicate TIF. Scale bars: 5 and 10 μm. (B) Mean number of TIF per nucleus in the airway epithelium of Ctrl or IPF lungs across the specified age ranges as indicated on the graph. On average, around 90 nuclei were analyzed per sample. Violin plots showing median, first and third quartile. Kruskal–Wallis test. ns: Not significant. (C) Percentage of nuclei showing at least 2 TIF in the airway epithelium of Ctrl or IPF lungs across the specified age ranges as indicated on the graph. Violin plots showing median, first and third quartile. Ordinary one‐way ANOVA test. (D) As in panel A, but including both airway epithelium and non‐airway tissues of the IPF lung. Scale bars: 5 and 10 μm. (E) Mean number of TIF per nucleus in the non‐airway tissues of Ctrl or IPF lungs across the specified age ranges as indicated on the graph. On average, around 100 nuclei were analyzed per sample. Kruskal–Wallis test. ns: Not significant. (F) qRT‐PCR analysis of *p16* expression in lung tissue samples from age‐matched Ctrl and IPF donors, as well as Ctrl donors aged 75 and older. *p16* mRNA levels were normalized to *ACTB* mRNA and to one IPF lung sample used as control. Ordinary one‐way ANOVA test. (G) p16 staining by immunohistochemistry and Alcian blue staining of fibrotic area in lung sample from a 60‐year‐old donor with IPF. HE, Hematoxylin–Eosin. Scale bar: 50 μm. (H) Representative images of SA‐β‐Gal activity staining in the airway epithelium of Ctrl or IPF lung samples from donors at the indicated age. Hematoxylin–Eosin (HE) staining is shown above. Scale bars: 50 μm.

Aging of the lung epithelium represents a major risk factor for IPF, a fatal disease characterized by cellular senescence and progressive lung scarring driven by excessive extracellular matrix deposition, with a prevalence that increases markedly after the age of 75 (Hewitt et al. 2025; Schneider et al. 2021). Our analyses revealed an increase in hallmarks of airway epithelial senescence in individuals older than 75 years. We therefore next compared age‐associated airway epithelial senescence in the lung with the senescence profile observed in IPF lungs. To address this, we compared TIF in lung samples from IPF patients aged 53–66 (*n* = 12), age‐matched controls (aged 56–63, *n* = 8), and older controls aged 75 and above (*n* = 8) (Table S1). Although the number of IPF lung samples analyzed was limited, TIF abundance in the airway epithelial tissue from IPF lungs (median of 2.2 TIF/nucleus) was approximately fivefold higher than in age‐matched controls (median of 0.4 TIF/nucleus) (Figures 2A,B and S2A). This level, however, was comparable to that observed in control lungs from donors aged 75 and above (Figures 2A,B and S2A). Similar results were obtained when comparing the percentage of cells containing at least two TIF per nucleus (Figure 2C). Previous work reported shorter telomere length in Alveolar type 2 (AT2) cells of IPF patients (Snetselaar et al. 2017). As AT2 cell dysfunction may be an important driver of IPF (Katzen and Beers 2020), we next tested whether AT2 alveolar cells may show distinct TIF positivity in both aged and IPF lungs. However, using combined telomeric FISH and immunofluorescence for γH2AX and pro‐SPC to specifically identify AT2 cells in IPF lungs, we found that the frequency of TIF‐positive cells was not significantly different between AT2 and non‐AT2 alveolar cells, nor between AT2 alveolar cells and airway epithelial cells (Figure S2B,C). Likewise, the frequency of TIF‐positive AT2 alveolar cells did not differ significantly between aged donors and donors with IPF, consistent with the results obtained in airway epithelial cells (Figure S2B,C). These results align with previous findings showing that, in IPF lungs, both bronchial epithelial cells and alveolar compartments exhibit comparable positivity for senescence markers (Schafer et al. 2017).

The “second hit” hypothesis posits that external insults to an already compromised, aging lung epithelium contribute to the onset of IPF (Hewitt et al. 2025). Our findings indicate that TIF may be involved in establishing this age‐related epithelial susceptibility. However, since TIF levels were comparable between lungs from IPF donors and age‐matched controls (≥ 75 years) with markedly different degrees of cellular senescence, TIF alone appears insufficient to drive the extensive senescence observed in IPF. This is supported by significantly elevated *p16* mRNA levels in IPF lungs (Figure 2F), and strong p16 protein staining in epithelial cells near fibrotic regions, marked by Alcian blue, consistent with previous studies (Minagawa et al. 2011; Schafer et al. 2017) (Figures 2G and S2D). The heightened senescence observed in IPF is further supported by a concurrent increase in SA‐β‐Gal activity within the epithelial layer (Figure 2H).

Collectively, these findings indicate that the rise in TIF frequency occurs only at a very advanced age, specifically within the lung epithelial cells of donors aged 75 and older, and support a potential causal role for TIF in IPF pathogenesis. Although additional IPF lung samples will be required to confirm these observations, it is likely that further external insults are required to initiate the pronounced cellular senescence observed in the IPF lung epithelium, characterized by elevated p16 expression and SA‐β‐Gal activity.

### 3.2 Telomere Shortening Without Major TIF Induction in Aging Nasal Epithelial Cells

**FIGURE 3:**
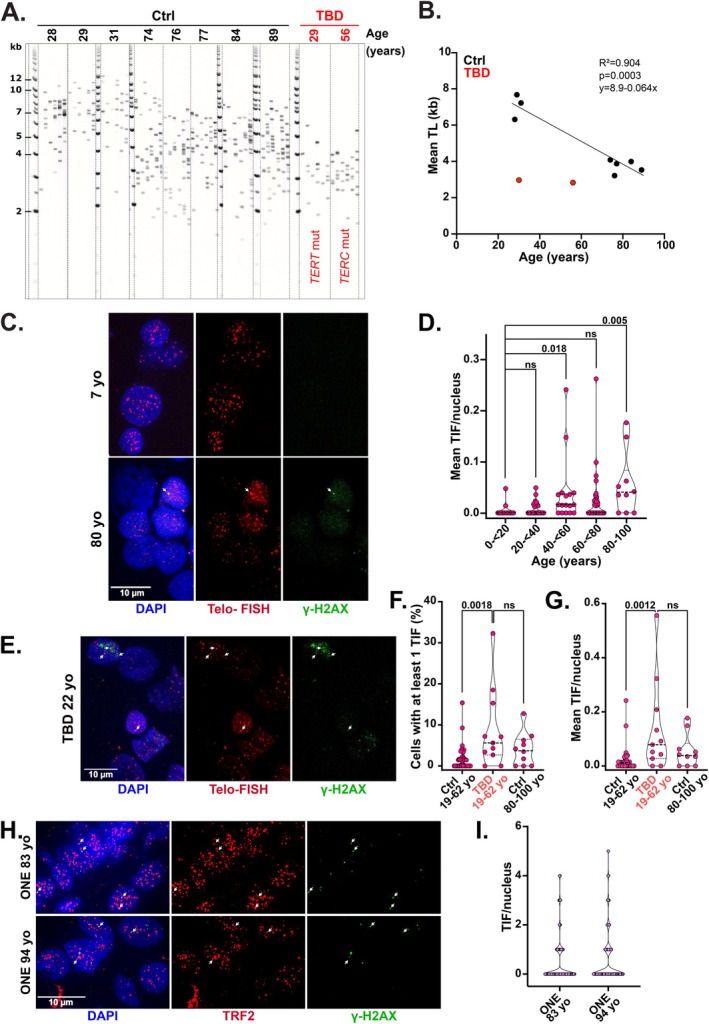
Telomere shortening without major TIF induction in aging nasal epithelial cells. (A) STELA analysis of TL in nasal brushing samples from donors without (Ctrl) or with telomere biology disorder (TBD). The age of the donors is indicated. Ladder (kb). (B) Linear regression analysis corresponding to panel A using nasal brushing samples from the Ctrl donors (black dots). Red dots represent TBD donors. (C) Representative images of TIF analysis in nasal brushing samples from Ctrl donors aged 7 and 80 years. Telomeres are detected by FISH with a telomeric probe (Telo‐FISH, red) and DNA damage is detected with an antibody against ɣ‐H2AX (green). DNA is stained with DAPI (blue). Arrows indicate TIF. Scale bar: 10 μm. (D) Mean number of TIF per nucleus in the nasal brushing samples of Ctrl donors across the specified age ranges. On average, around 65 nuclei were analyzed per sample. Violin plots showing median, first and third quartile. Kruskal–Wallis tests. ns: Not significant. (E) As in panel C, but in the nasal brushing sample from a 22‐year‐old donor with TBD. Arrows indicate TIF. Scale bar: 10 μm. (F) Percentage of nuclei showing at least one TIF per nucleus based on the analysis described in panel F. Violin plots showing median, first and third quartile. Kruskal‐Wallis tests. ns: Not significant. (G) Mean number of TIF per nucleus in the nasal brushing samples from Ctrl and TBD donors as indicated on the graph. On average, around 80 nuclei were analyzed per sample. Violin plots showing median, first and third quartile. Kruskal–Wallis tests. ns: Not significant. (H) Representative images of TIF analysis in the ONE tissue of two donors aged 83 and 94 years. Telomeres are detected with an antibody against TRF2 telomere‐binding protein (red) and DNA damage is detected with an antibody against ɣ‐H2AX (green). DNA is stained with DAPI (blue). Arrows indicate TIF. Scale bar: 10 μm. (I) Number of TIF per nucleus in the ONE samples from panel H. A total of 85 and 101 nuclei were counted per sample, respectively.

Cellular aging progresses along distinct, organ‐specific trajectories and exhibits temporal variability (Schaum et al. 2020). To investigate whether senescence markers in the upper respiratory tract mirror those observed in the aging lung epithelium, we developed a minimally invasive nasal brushing method to collect epithelial cells from healthy donors ranging in age from 2 to 97 years (Table S2 and Figure S3). First, we assessed whether TL decreases with age in nasal epithelial cells. Using STELA analysis (Baird et al. 2003) on samples from young (*n* = 3) and older (*n* = 5) donors, we estimated an age‐related TL attrition rate of approximately 64 ± 8 base pairs per year (Figure 3A,B, black). This value is comparable to the 59–60 bp/year telomere shortening rate observed in the colon and the esophagus, with high regenerative activity (Takubo et al. 2010). Nasal brushing samples from two individuals with telomere biology disorders (TBD), carrying mutations in either the *TERC* (age 56) or *TERT* (age 29) subunit of telomerase, revealed markedly short telomeres, comparable to the average TL observed in nasal epithelial cells from control individuals aged 75 and older (Figure 3A,B). Collectively, these findings support the utility of nasal epithelial cells as a valid model for studying telomere dynamics during aging.

Analysis of TIF formation in nasal epithelial cells revealed a modest age‐associated increase in TIF levels (Figure 3C,D). Despite this trend, the median number of TIF per nucleus remained very low, rising only slightly from 0 to 0.04 across age groups (Figure 3D), indicating a generally low TIF abundance in this tissue. Nasal epithelial cells from TBD patients aged 19–62 displayed a higher TIF burden compared to age‐matched controls, but levels were similar to those seen in the older control group aged 80 and above (Figure 3E–G). This supports the notion of overall low TIF abundance in nasal epithelium. Additionally, our finding that the average number of γ‐H2AX foci in samples from older individuals did not significantly increase (Figure S4) argues against the possibility of a bias in TIF detection resulting from telomeres being too short to be visualized by FISH. In the olfactory neuroepithelium—located near the nasal epithelium—and sampled from two deceased donors aged 83 and 94 years, TIF levels were also extremely low (median of 0) (Figure 3H,I). These results contrast with the increased TIF abundance observed in the aging lung epithelium, highlighting the previously reported variability in age‐dependent TIF (Jeyapalan et al. 2007).

These findings indicate that, although TL decreases with age, nasal epithelial cells from healthy individuals show minimal accumulation of TIF.

### 3.3 Early Proteostasis Disruption in Nasal Epithelial Cells

As no single marker can definitively identify senescent cells (Gorgoulis et al. 2019), and given the minimal accumulation of TIF observed in aged nasal epithelial cells, we proceeded to examine additional senescence markers in the nasal brushing samples.

**FIGURE 4:**
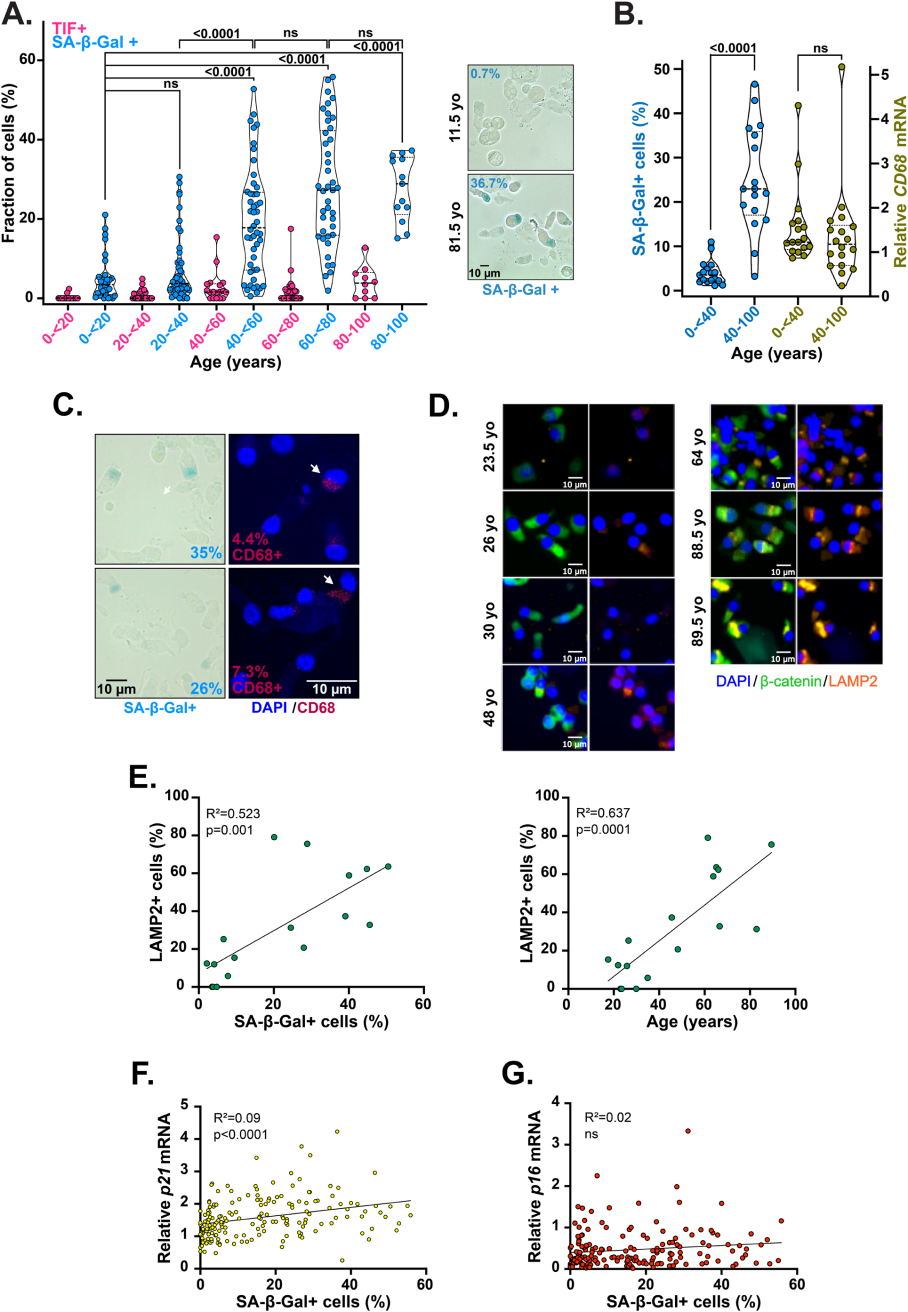
Early proteostasis disruption in nasal epithelial cells. (A) Percentage of SA‐β‐Gal‐positive cells (SA‐β‐Gal+, blue) in nasal brushing samples and percentage of nuclei showing at least one TIF per nucleus (TIF+, pink) across the specified age ranges. For SA‐β‐Gal staining, on average, around 500 nuclei were analyzed per sample. Violin plots showing median, first and third quartile. Kruskal–Wallis tests. ns: Not significant. Right: Representative images of SA‐β‐Gal staining from two donors aged 11.5 and 81.5 years. Scale bar: 10 μm. (B) Percentage of SA‐β‐Gal‐positive cells (blue) and relative *CD68* mRNA expression (khaki) in a subset of nasal brushing samples from donors below or above 40 years old. *CD68* mRNA levels were normalized to *ACTB* mRNA and to a reference sample. Mann–Whitney tests. ns: Not significant. (C) Left: Representative images of SA‐β‐Gal staining in two nasal brushing samples with the indicated percentages of SA‐β‐Gal+ cells. Right: CD68 detection by immunofluorescence (red) on the same nasal brushing samples. DNA is stained with DAPI (blue). The percentage of CD68‐positive cells (arrows) is indicated. Scale bar: 10 μm. (D) Representative images of LAMP2 staining (orange) in nasal brushing samples from donors at the indicated ages. Cytoplasm is detected by β‐catenin staining (green). DNA is stained with DAPI (blue). Scale bar: 10 μm. (E) Linear regression analyses showing the percentage of LAMP2+ cells plotted against either SA‐β‐Gal+ cells (left) or donor age (right). (F, G) Linear regression analyses showing *p21* (F) or *p16* (G) expression levels in nasal brushing samples plotted against the percentage of SA‐β‐Gal+ cells. ns, not significant.

The colorimetric assay for SA‐β‐Gal activity (Dimri et al. 1995), which reflects the elevated lysosomal content characteristic of senescent cells undergoing proteotoxic stress, remains the most widely used approach for identifying senescent cells both in vitro and in vivo (Dimri and Dimri 2024). Using this method, we found that the proportion of SA‐β‐Gal‐positive cells (SA‐β‐Gal+) increases with age, showing a significant rise in individuals aged 40 and above, followed by a plateau around the age of 60 (Figure 4A). In the oldest age group—individuals over 80 years—approximately 30% of the collected cells were SA‐β‐Gal+ (Figure 4A). Notably, this increase in SA‐β‐Gal positivity occurred well before the modest rise in TIF frequency observed in the oldest individuals (Figure 4A), indicating that SA‐β‐Gal expression does not result from TIF induction.

One limitation of using SA‐β‐Gal as a senescence marker is its tendency to also stain macrophages (Sharpless and Sherr 2015). To rule out the possibility that the observed increase in SA‐β‐Gal positivity in nasal brushing samples was due to macrophages, we measured *CD68* mRNA levels, a recognized macrophage marker. Despite the significant elevation in SA‐β‐Gal+ cells, there was no statistically significant difference in *CD68* mRNA expression between the 0‐ < 40 and 40–100 years age groups (Figure 4B). This was confirmed by staining two independent nasal brushing samples, which revealed SA‐β‐Gal positivity clearly distinct from the proportion of CD68‐positive cells (Figure 4C).

Earlier studies proposed that elevated SA‐β‐Gal activity in senescent fibroblasts reflects increased lysosomal content and accumulation of degradative autolysosomes (Gerland et al. 2003). Accordingly, the rise in SA‐β‐Gal positivity in nasal epithelial cells around age 40 may indicate an early impairment in proteostasis. To strengthen this observation, we employed an orthogonal method by staining samples with an antibody against LAMP2, lysosomal‐associated membrane protein, to assess lysosome enlargement. Cells were considered LAMP2+ if they showed intense cytoplasmic staining (Figure 4D). Consistent with SA‐β‐Gal staining and prior findings of increased LAMP2 expression in senescent melanoma cells (Rovira et al. 2022), the proportion of LAMP2+ nasal epithelial cells rose significantly with donor age and showed a strong correlation with SA‐β‐Gal positivity (Figure 4E).

Finally, we examined whether the increased SA‐β‐Gal positivity in aging nasal epithelial cells was linked to elevated expression of the senescence markers ***p21*** or ***p16***. While ***p21*** mRNA showed a moderate correlation with SA‐β‐Gal positivity (Figure 4F), ***p16*** expression levels did not correlate with SA‐β‐Gal positivity in nasal epithelial cells (Figure 4G).

Together, these results point to an association between aging of nasal epithelial cells and proteostatic stress.

### 3.4 SA‐β‐Gal Activity in Nasal Epithelial Cells Negatively Correlates With Olfactory Function

**FIGURE 5:**
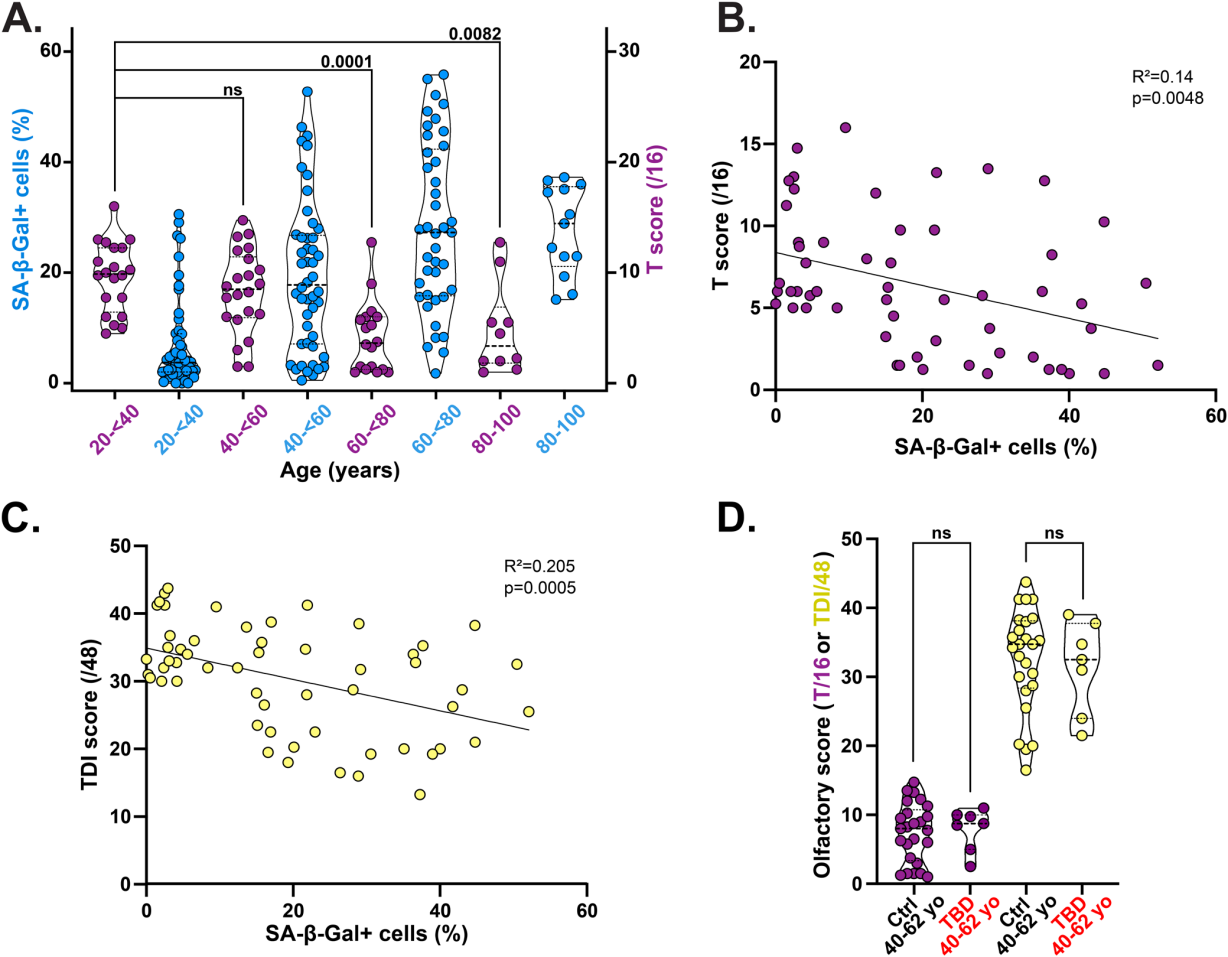
SA‐β‐Gal activity in nasal epithelial cells negatively correlates with olfactory function. (A) Percentage of SA‐β‐Gal+ cells (blue, same as Figure 4A) in nasal brushing samples and Threshold (T) olfactory score (mauve) in donors across the specified age ranges. Violin plots showing median, first and third quartile. Kruskal–Wallis tests. ns, not significant. (B) Linear regression analysis of T score relative to the percentage of SA‐β‐Gal+ nasal brushing cells in a total of 75 donors aged 18–97 years. (C) As in panel B, but for the TDI (Threshold + Discrimination + Identification) olfactory score. (D) T (mauve) or TDI (yellow) olfactory scores in age‐matched Ctrl and TBD patients. Violin plots showing median, first and third quartile. Unpaired *t* tests. ns, not significant.

Olfactory function gradually declines beginning in early adulthood, with the most marked reduction observed in odor threshold sensitivity, as opposed to odor discrimination or identification (Oleszkiewicz et al. 2019). Odor discrimination and identification rely on both the olfactory nasal epithelium and central brain regions such as the entorhinal cortex and hippocampus to process the information (Kjelvik et al. 2012). Conversely, odor threshold is more directly influenced by olfactory nasal epithelium function (Hummel et al. 2017). To investigate whether aging of the olfactory nasal epithelium contributes to olfactory decline, we assessed odor threshold in healthy individuals aged 18 to 97. Using the Sniffin' Sticks test, we found that declining threshold scores with age were associated with increased SA‐β‐Gal positivity in nasal epithelial cells (Figure 5A,B), suggesting a potential link between proteostasis loss in these aging cells and olfactory decline. Since the olfactory epithelium supports the maintenance and function of olfactory neurons, its aging is also likely to impair overall olfactory performance. In line with this, the total TDI score showed a negative correlation with SA‐β‐Gal positivity in nasal epithelial cells (Figure 5C). Interestingly, TBD patients did not exhibit any premature olfactory deficit (Figure 5D), further supporting the idea that telomere dysfunction is not a driver of aging in the olfactory nasal epithelium.

Taken together, these findings indicate that impaired proteostasis in the aging olfactory nasal epithelium contributes to the age‐related decline in olfactory function.

## 4 Discussion

In patients with TBD, telomere dysfunction is a well‐established and widely accepted contributor to accelerated cellular and tissue aging, as well as the early onset of age‐related diseases (Revy et al. 2023; Rossiello et al. 2022). In contrast, during normal aging, only low levels of damaged telomeres have been reported in various tissues of aging baboons, including skin, hippocampal neurons, and liver hepatocytes (Fumagalli et al. 2012; Herbig et al. 2006; Jeyapalan et al. 2007), as well as in skin biopsies from elderly human donors (Victorelli et al. 2019). In both baboon and human tissues, the average TIF burden per nucleus remains modest compared to the elevated levels observed during replicative senescence in vitro (Jeyapalan et al. 2007; Victorelli et al. 2019). While early studies in telomerase‐deficient mice indicated that just one or a few critically short telomeres could be sufficient to trigger cellular senescence in vivo (Hemann et al. 2001), the extent to which TIF contributes to physiological aging in primates remains unresolved. Given the heterogeneity of cellular senescence (Cohn et al. 2023), it is also likely that the contribution of TIF to senescence is not universal across cell types or tissues.

Patients with TBD exhibit a strong predisposition to early‐onset IPF, supporting a causal link between telomere dysfunction in the lung epithelium and IPF pathogenesis (Revy et al. 2023; Rossiello et al. 2022). In contrast, among individuals without TBD, IPF prevalence rises sharply only after the age of 75 (Hewitt et al. 2025; Schneider et al. 2021). Whether this is associated with TIF accumulation in the lung epithelium remains unclear, as most studies to date have focused on lung tissues from individuals younger than 80 years old. In this study, we show that TIF frequency in the airway epithelium rises significantly in donors aged 75 and older, reaching levels comparable to those observed in IPF lungs. Although we acknowledge that the limited number of lung samples analyzed represents a limitation of our study, these findings suggest a potential causal link between TIF accumulation and the development of IPF during physiological aging. The “second hit” hypothesis proposes that IPF arises from additional exogenous insults acting on an already vulnerable, aging lung epithelium (Hewitt et al. 2025). It is therefore plausible that TIF induction contributes to this vulnerability, and that further environmental exposures, such as cigarette smoke, intensify cellular senescence and drive fibrosis. Consistent with our previous findings showing that short telomeres elevate the risk of severe COVID‐19 (Froidure et al. 2020), the late‐life increase in TIF within the lung epithelium may also possibly contribute to the heightened vulnerability to COVID‐19‐related mortality, which is reported to be approximately 50 times higher in individuals aged 80 and above compared to those aged 18–39 (Williamson et al. 2020).

Age‐related alterations in the nasal epithelium have been proposed to contribute to olfactory decline (Doty and Kamath 2014; Kondo et al. 2020; Mobley et al. 2014). Although the decline in odor identification function may be partly attributed to broader age‐related deterioration in central nervous system functions, which we did not address in this study, severe but transient damage to the olfactory epithelium, independent of injury to the central nervous system, has been linked to anosmia, as observed during SARS‐CoV‐2 infection (Bryche et al. 2020). Similarly, cumulative environmental insults to the olfactory epithelium have been proposed as a contributing factor to age‐related olfactory decline (Attems et al. 2015). While the exact mechanisms by which epithelial damage contributes to age‐associated olfactory loss remain unclear, they may involve functional impairments such as reduced ciliary beat frequency and prolonged nasal mucociliary clearance, both of which have been reported in individuals over the age of 40 (Ho et al. 2001). Aging of the upper respiratory tract, however, has been relatively understudied, and whether TIF play any role in this part of the respiratory tract remains unexplored. Our findings suggest that TIF are unlikely to be the primary drivers of age‐related senescence in nasal epithelial cells. Instead, we propose that this nasal epithelium senescence results from a gradual loss of proteostasis beginning after the age of 40. This is consistent with recent findings suggesting a widespread breakdown of proteostasis in aging human tissues, with a notable acceleration in aging processes typically occurring around the age of 50 (Ding et al. 2025). Proteostasis, the maintenance of a balanced system for protein synthesis, folding, trafficking, and degradation, can be disrupted under conditions such as oxidative stress (Korovila et al. 2017). Due to their direct exposure to the external environment, nasal epithelial cells are particularly vulnerable to oxidative stress (Yamamoto et al. 2023), which may lead to proteostatic imbalance. Recently, widespread amyloid protein aggregation was identified as a hallmark of aging across multiple human organs (Ding et al. 2025); however, whether similar protein aggregation occurs in the nasal epithelium remains to be determined. In support of this possibility, pathological accumulation of amyloid‐β and PHF‐tau has been reported in the olfactory epithelium of aged individuals, although the absence of tissue from younger donors in that study limits direct comparison (Arnold et al. 2010). Notably, hallmarks of aging in the nasal epithelium appeared distinct from those observed in the lung as, consistent with earlier reports (Minagawa et al. 2011), we did not detect a consistent age‐related increase in SA‐β‐Gal staining in the epithelium of aging lungs.

Overall, our findings suggest that TIF are unlikely to serve as universal drivers of cellular senescence, which appears to arise through diverse mechanisms across different human cell types (Cohn et al. 2023). Future research involving human tissues, biopsies, or ex vivo cellular analyses will be essential to more accurately elucidate the mechanisms of cellular senescence in the human body, as traditional in vitro models of replicative senescence do not fully capture the complexity of senescence as it occurs in vivo.

## Author Contributions

C.C., K.B., M.M., K.N., D.H., and Am.D. performed the experiments. S.E.V., L.D.S., A.V., W.W., J.V.S., and L.J.C. provided lung samples. D.M.B. supervised the STELA analyses. C.C. recruited the volunteers for nasal brushing with the help of An.D., A.F., and M.V.G. C.C. performed the nasal brushing and C.C. and K.B. performed the olfactory tests. C.H. contributed to nasal brushings and olfactory tests. C.C., K.B., and M.M. prepared the figures and participated in writing the manuscript. An.D. supervised the study and wrote the manuscript. All authors approved the final version of the manuscript.

## Funding

C.C. and Am.D. are supported by the Télévie‐FNRS (Belgium), K.B. and C.H. are supported by UCLouvain (ARC, Action de Recherche Concertée), M.M. is supported by UCLouvain and the FNRS, A.L. is supported by the de Duve Institute, Baird lab is funded by Cancer Research UK programme C17199/A29202 and the Wales Cancer Research Centre, A.F. is supported by a grant from the Fonds de Recherche Clinique, Cliniques universitaires Saint‐Luc, L.J.C. is funded by Research Council Flanders (FWO, 18E2B24N), An.D. is supported by the FNRS. The work conducted in An.D.'s lab was supported by Salus Sanguinis, the King Baudouin Foundation (Maurange Fund), the FNRS, Télévie‐FNRS, UCLouvain, and the de Duve Institute. We gratefully acknowledge their ongoing support.

## Conflicts of Interest

The authors declare no conflicts of interest.

## Supporting information


**Figure S1:** Senescence markers in aging human lungs. (A) Representative images of TIF analysis in the airway epithelium from Ctrl donors at the indicated age. Telomeres are detected with an antibody against TRF2 telomere‐binding protein (red) and DNA damage is detected with an antibody against ɣ‐H2AX (green). DNA is stained with DAPI (blue). Scale bar: 10 μm. (B) qRT‐PCR analysis of *p16* expression in lung samples of donors across the specified age ranges. *p16* mRNA levels were normalized to *ACTB* mRNA and to one IPF lung sample used as control. Violin plots showing median, first and third quartile. Unpaired *t* test. ns: not significant. (C) Linear regression analysis of *p16* expression levels in lung samples relative to donor age.

**Figure S2:** Hallmarks of cellular senescence in aged and IPF lungs. (A) Representative images of TIF analysis in the airway epithelium of IPF lungs from donors at the indicated age. Telomeres are detected with an antibody against TRF2 telomere‐binding protein (red) and DNA damage is detected with an antibody against ɣ‐H2AX (green). DNA is stained with DAPI (blue). Scale bar: 10 μm. (B) Representative images of TIF analysis in the airway and alveolar epithelium of lung samples from a 75‐year‐old control donor and a 60‐year‐old IPF donor. Telomeres are detected with a telomeric FISH probe (red), DNA damage is detected with an antibody against ɣ‐H2AX (green) and AT2 cells are detected with an antibody against pro‐SPC (white). DNA is stained with DAPI (blue). Scale bars: 10 and 5 μm. (C) Mean frequency of AT2 and non‐AT2 cells in the alveolar epithelium of aging lungs (75–83 years old, *n* = 4) and IPF lungs (60–61 years old, *n* = 4) containing at least one TIF. On average, approximately 60 AT2 nuclei and 140 non‐AT2 nuclei were analyzed per sample in the alveolar epithelium. For the airway epithelium of IPF lungs, the mean frequency of TIF‐positive cells was determined from approximately 50 nuclei per sample. Violin plots showing median, first and third quartile. One‐way ANOVA. ns: not significant. (D) Representative images of p16 staining by immunohistochemistry in IPF lung samples from two 60‐year‐old donors. HE: Hematoxylin–Eosin. Scale bar: 50 μm.

**Figure S3:** Age distribution of control donors providing nasal brushing samples.

**Figure S4:** No significant increase in total ɣ‐H2AX signals in aging human nasal epithelial cells. Mean number of total ɣ‐H2AX foci per nucleus in the nasal brushing samples of donors across the specified age ranges. On average, around 65 nuclei were analyzed per sample. Violin plots showing median, first and third quartile. Kruskal‐Wallis test. ns: not significant.

**Table S1:** Lung samples used in this study.

**Table S2:** Volunteers for nasal brushing and/or olfactory tests.

**Table S3:** Antibodies used in this study. All antibodies were used in IF experiments.

**Table S4:** Primers used in this study for qRT‐PCR. All primers were obtained from Eurogentec (Belgium).

## Data Availability

The data that support the findings of this study are available on request from the corresponding author. The data are not publicly available due to privacy or ethical restrictions.
