# Supplementary figures and images for: Telomere Dysfunction and Proteostasis Decline Define Distinct Pathways of Cellular Senescence in the Human Respiratory Tract
Source: Aging Cell. 2026 Apr 20;25(5):e70512. doi: 10.1111/acel.70512 (PMC13096579; doi:10.1111/acel.70512)

**A.**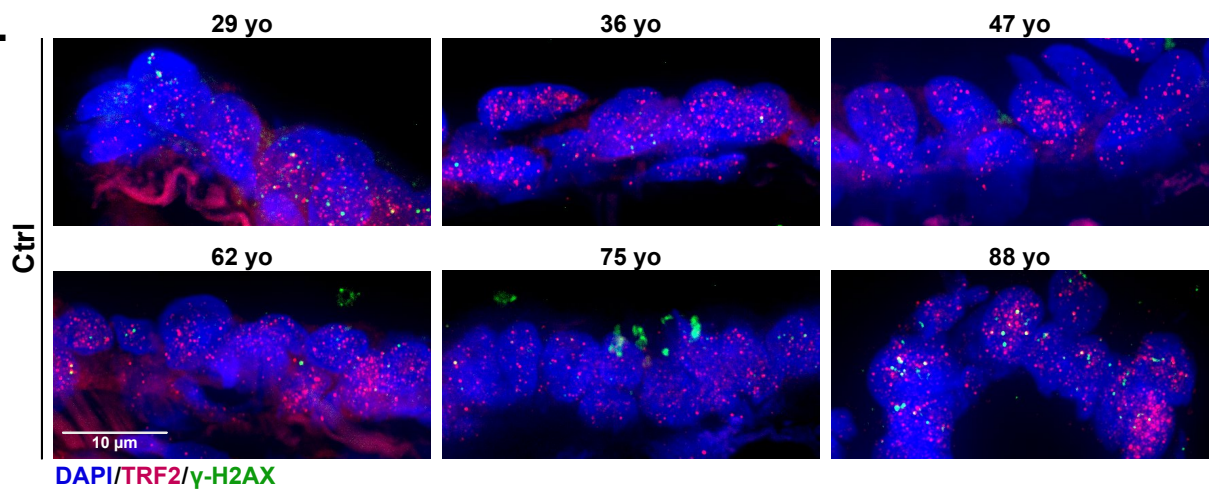**B.**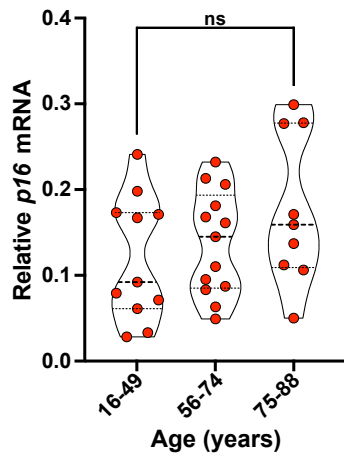**C.**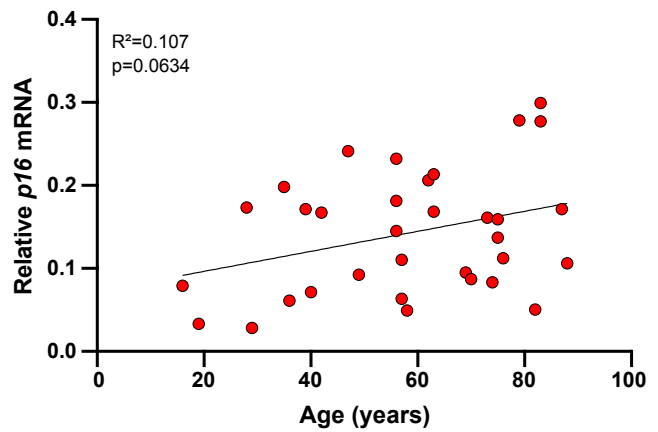

Supplement: Supplementary file 1 — Figure S1: Senescence markers in aging human lungs. (A) Representative images of TIF analysis in the airway epithelium from Ctrl donors at the indicated age. Telomeres are detected with an antibody against TRF2 telomere‐binding protein (red) and DNA damage is detected with an antibody against ɣ‐H2AX (green). DNA is stained with DAPI (blue). Scale bar: 10 μm. (B) qRT‐PCR analysis of p16 expression in lung samples of donors across the specified age ranges. p16 mRNA levels were normalized to ACTB mRNA and to one IPF lung sample used as control. Violin plots showing median, first and third quartile. Unpaired t test. ns: not significant. (C) Linear regression analysis of p16 expression levels in lung samples relative to donor age. [file ACEL-25-e70512-s002.pdf]

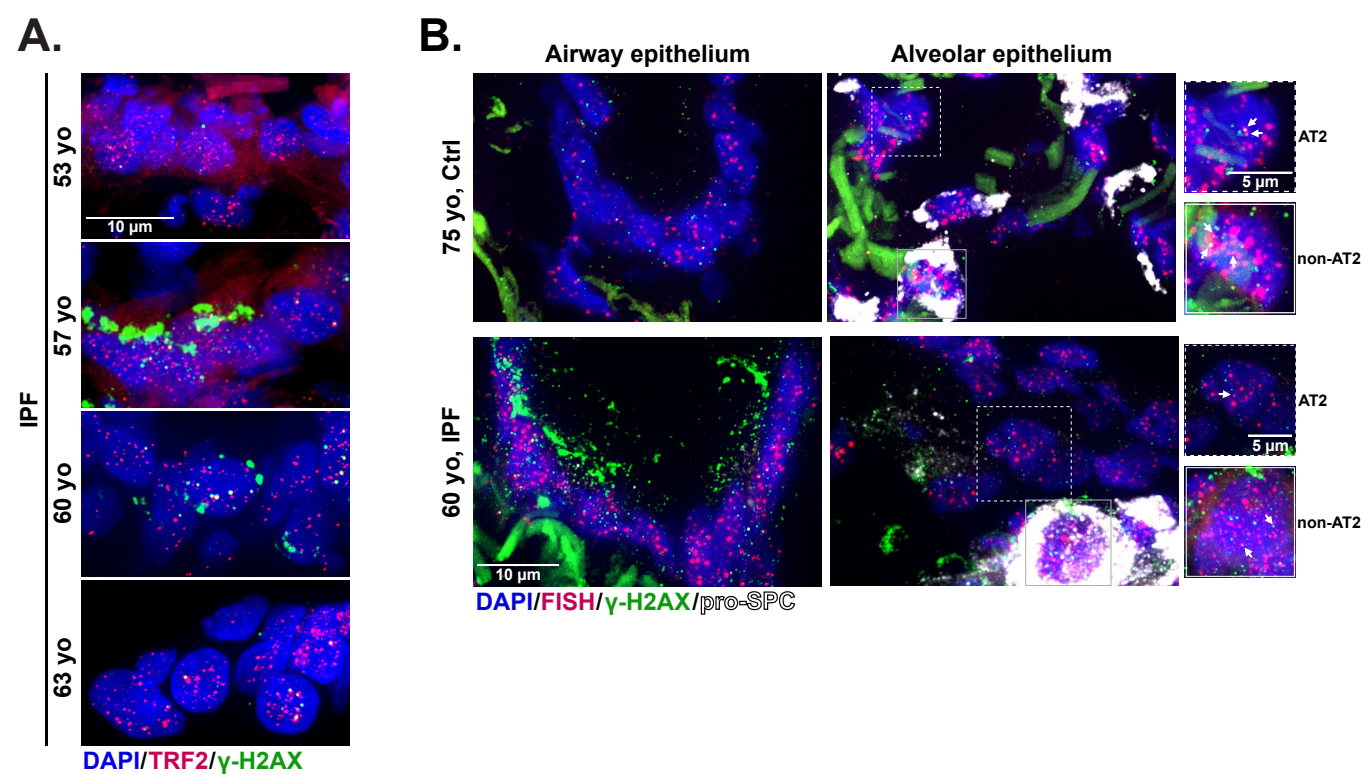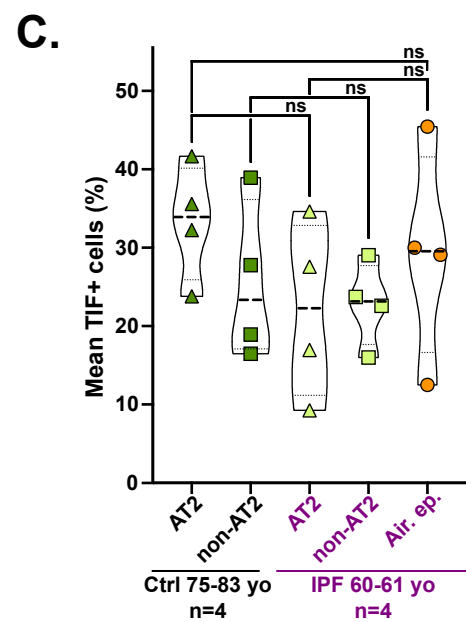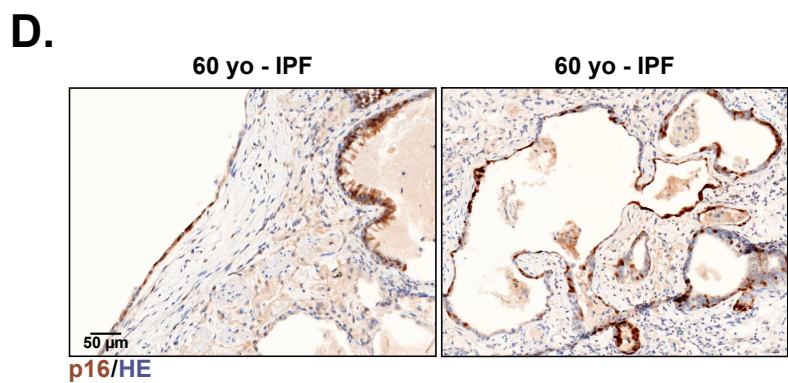

Supplement: Supplementary file 2 — Figure S2: Hallmarks of cellular senescence in aged and IPF lungs. (A) Representative images of TIF analysis in the airway epithelium of IPF lungs from donors at the indicated age. Telomeres are detected with an antibody against TRF2 telomere‐binding protein (red) and DNA damage is detected with an antibody against ɣ‐H2AX (green). DNA is stained with DAPI (blue). Scale bar: 10 μm. (B) Representative images of TIF analysis in the airway and alveolar epithelium of lung samples from a 75‐year‐old control donor and a 60‐year‐old IPF donor. Telomeres are detected with a telomeric FISH probe (red), DNA damage is detected with an antibody against ɣ‐H2AX (green) and AT2 cells are detected with an antibody against pro‐SPC (white). DNA is stained with DAPI (blue). Scale bars: 10 and 5 μm. (C) Mean frequency of AT2 and non‐AT2 cells in the alveolar epithelium of aging lungs (75–83 years old, n = 4) and IPF lungs (60–61 years old, n = 4) containing at least one TIF. On average, approximately 60 AT2 nuclei and 140 non‐AT2 nuclei were analyzed per sample in the alveolar epithelium. For the airway epithelium of IPF lungs, the mean frequency of TIF‐positive cells was determined from approximately 50 nuclei per sample. Violin plots showing median, first and third quartile. One‐way ANOVA. ns: not significant. (D) Representative images of p16 staining by immunohistochemistry in IPF lung samples from two 60‐year‐old donors. HE: Hematoxylin–Eosin. Scale bar: 50 μm. [file ACEL-25-e70512-s004.pdf]

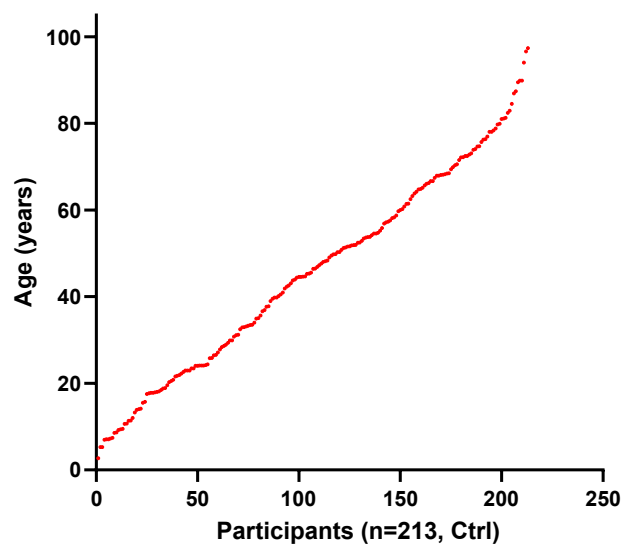

Supplement: Supplementary file 3 — Figure S3: Age distribution of control donors providing nasal brushing samples. [file ACEL-25-e70512-s001.pdf]

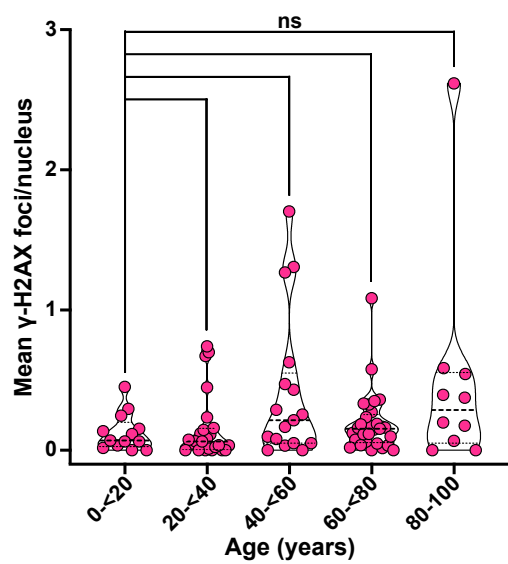

Supplement: Supplementary file 4 — Figure S4: No significant increase in total ɣ‐H2AX signals in aging human nasal epithelial cells. Mean number of total ɣ‐H2AX foci per nucleus in the nasal brushing samples of donors across the specified age ranges. On average, around 65 nuclei were analyzed per sample. Violin plots showing median, first and third quartile. Kruskal‐Wallis test. ns: not significant. [file ACEL-25-e70512-s006.pdf]
