# Supplementary material for: Telomere Dysfunction and Proteostasis Decline Define Distinct Pathways of Cellular Senescence in the Human Respiratory Tract
Source: Aging Cell. 2026 Apr 20;25(5):e70512. doi: 10.1111/acel.70512 (PMC13096579; doi:10.1111/acel.70512)
Supplement: Supplementary file 5 — Table S1: Lung samples used in this study. [file ACEL-25-e70512-s007.docx]

**Supplementary Table 1. Lung samples used in this study.**

| **Sample** | **Age (years)** | **Sex** | **Comment** |
| --- | --- | --- | --- |
| CTRL 1 | 16 | M | Unused : artery rupture |
| CTRL 2 | 19 | M | Declined : oedema |
| CTRL 3 | 28 | F | Peripheral wedge biopsy prior to implantation |
| CTRL 4 | 29 | M | Declined : contusion |
| CTRL 5 | 35 | M | Peripheral wedge biopsy prior to implantation |
| CTRL 6 | 36 | M | Declined : infection |
| CTRL 7 | 39 | M | Used for lobar transplant |
| CTRL 8 | 40 | F | Used for lobar transplant |
| CTRL 9 | 42 | M | Peripheral wedge biopsy prior to implantation |
| CTRL 10 | 47 | F | Used for lobar transplant |
| CTRL 11 | 49 | F | Peripheral wedge biopsy prior to implantation |
| CTRL 12 | 56 | M | Declined : infection |
| CTRL 13 | 56 | M | Used for single lung transplant |
| CTRL 14 | 56 | M | Peripheral wedge biopsy prior to implantation |
| CTRL 15 | 57 | F | Declined : embolus |
| CTRL 16 | 57 | M | Declined : embolus |
| CTRL 17 | 58 | M | Declined : infection |
| CTRL 18 | 62 | F | Declined : infection |
| CTRL 19 | 63 | M | Declined : contusion |
| CTRL 20 | 63 | F | Peripheral wedge biopsy prior to implantation |
| CTRL 21 | 69 | F | Declined : embolus |
| CTRL 22 | 70 | F | Peripheral wedge biopsy prior to implantation |
| CTRL 23 | 73 | M | Peripheral wedge biopsy prior to implantation |
| CTRL 24 | 74 | M | Declined : contusion |
| CTRL 25 | 75 | M | Declined : gastric tumor |
| CTRL 26 | 75 | F | Peripheral wedge biopsy prior to implantation |
| CTRL 27 | 76 | M | Peripheral wedge biopsy prior to implantation |
| CTRL 28 | 79 | F | Declined : pleuritis on contralateral lung |
| CTRL 29 | 82 | M | Declined : fragile lung |
| CTRL 30 | 83 | M | Declined : secretions |
| CTRL 31 | 83 | F | Peripheral wedge biopsy prior to implantation |
| CTRL 32 | 87 | F | Peripheral wedge biopsy prior to implantation |
| CTRL 33 | 88 | M | Peripheral wedge biopsy prior to implantation |
|  |  |  |  |
| IPF 1 | 53 | M | Familial IPF |
| IPF 2 | 55 | M | Familial IPF |
| IPF 3 | 57 | M | Sporadic IPF |
| IPF 4 | 60 | M | Familial IPF |
| IPF 5 | 60 | M | Sporadic IPF |
| IPF 6 | 61 | M | Familial IPF |
| IPF 7 | 61 | M | Familial IPF |
| IPF 8 | 61 | M | Sporadic IPF |
| IPF 9 | 62 | F | Familial IPF |
| IPF 10 | 62 | M | Sporadic IPF |
| IPF 11 | 63 | F | Sporadic IPF |
| IPF 12 | 66 | F | Sporadic IPF |
