# Supplementary material for: Telomere Dysfunction and Proteostasis Decline Define Distinct Pathways of Cellular Senescence in the Human Respiratory Tract
Source: Aging Cell. 2026 Apr 20;25(5):e70512. doi: 10.1111/acel.70512 (PMC13096579; doi:10.1111/acel.70512)
Supplement: Supplementary file 6 — Table S2: Volunteers for nasal brushing and/or olfactory tests. [file ACEL-25-e70512-s005.docx]

**Supplementary Table 2. Volunteers for nasal brushing and/or olfactory tests.**

|  | **Age (years)** | **Sex** | **Nasal brushing** | **Olfactory test** |
| --- | --- | --- | --- | --- |
| CTRL | 2,71 | F | YES | NO |
| CTRL | 5,27 | M | YES | NO |
| CTRL | 5,28 | M | YES | NO |
| CTRL | 6,96 | M | YES | NO |
| CTRL | 7,06 | M | YES | NO |
| CTRL | 7,06 | M | YES | NO |
| CTRL | 7,25 | M | YES | NO |
| CTRL | 7,43 | M | YES | NO |
| CTRL | 8,56 | M | YES | NO |
| CTRL | 8,69 | M | YES | NO |
| CTRL | 9,20 | M | YES | NO |
| CTRL | 9,40 | M | YES | NO |
| CTRL | 9,47 | F | YES | NO |
| CTRL | 10,66 | F | YES | NO |
| CTRL | 10,70 | F | YES | NO |
| CTRL | 11,36 | M | YES | NO |
| CTRL | 11,41 | F | YES | NO |
| CTRL | 12,01 | F | YES | NO |
| CTRL | 13,28 | F | YES | NO |
| CTRL | 13,85 | F | YES | NO |
| CTRL | 14,01 | M | YES | NO |
| CTRL | 14,15 | F | YES | NO |
| CTRL | 15,51 | F | YES | NO |
| CTRL | 15,73 | M | YES | NO |
| CTRL | 17,53 | F | YES | NO |
| CTRL | 17,68 | F | YES | NO |
| CTRL | 17,79 | M | YES | YES |
| CTRL | 17,80 | M | YES | YES |
| CTRL | 17,94 | M | YES | NO |
| CTRL | 18,01 | M | YES | NO |
| CTRL | 18,15 | F | YES | NO |
| CTRL | 18,49 | F | YES | NO |
| CTRL | 18,78 | F | YES | NO |
| CTRL | 18,93 | M | YES | NO |
| CTRL | 19,54 | M | YES | NO |
|  |  |  |  |  |
|  |  |  |  |  |
| CTRL | 20,26 | F | YES | NO |
| CTRL | 20,56 | F | YES | NO |
| CTRL | 20,83 | F | YES | NO |
| CTRL | 21,57 | M | YES | NO |
| CTRL | 21,74 | F | YES | NO |
| CTRL | 21,87 | F | NO | YES |
| CTRL | 21,91 | M | YES | NO |
| CTRL | 22,22 | F | NO | YES |
| CTRL | 22,26 | F | YES | NO |
| CTRL | 22,28 | F | NO | YES |
| CTRL | 22,32 | F | NO | YES |
| CTRL | 22,61 | M | YES | NO |
| CTRL | 22,94 | F | YES | NO |
| CTRL | 22,94 | F | YES | NO |
| CTRL | 22,97 | F | YES | NO |
| CTRL | 23,42 | F | YES | YES |
| CTRL | 23,47 | F | YES | NO |
| CTRL | 23,98 | M | NO | YES |
| CTRL | 24,04 | M | YES | NO |
| CTRL | 24,05 | F | YES | NO |
| CTRL | 24,08 | M | YES | NO |
| CTRL | 24,11 | F | YES | NO |
| CTRL | 24,12 | F | YES | YES |
| CTRL | 24,22 | F | YES | NO |
| CTRL | 24,35 | F | YES | NO |
| CTRL | 24,67 | M | NO | YES |
| CTRL | 25,12 | F | NO | YES |
| CTRL | 25,53 | F | NO | YES |
| CTRL | 25,79 | F | YES | YES |
| CTRL | 25,84 | F | YES | NO |
| CTRL | 26,08 | M | NO | YES |
| CTRL | 26,47 | F | YES | NO |
| CTRL | 26,57 | M | YES | NO |
| CTRL | 27,11 | F | YES | YES |
| CTRL | 27,80 | F | YES | NO |
| CTRL | 28,36 | F | YES | NO |
| CTRL | 28,64 | F | YES | NO |
| CTRL | 28,90 | M | YES | NO |
| CTRL | 28,91 | F | NO | YES |
| CTRL | 29,36 | F | YES | YES |
| CTRL | 29,89 | F | YES | NO |
| CTRL | 29,91 | F | YES | NO |
| CTRL | 30,03 | F | NO | YES |
| CTRL | 30,77 | M | YES | NO |
| CTRL | 31,07 | F | NO | YES |
| CTRL | 31,10 | M | YES | NO |
| CTRL | 31,21 | F | YES | NO |
| CTRL | 32,49 | M | YES | NO |
| CTRL | 32,91 | F | YES | NO |
| CTRL | 32,98 | M | YES | YES |
| CTRL | 33,14 | M | YES | NO |
| CTRL | 33,32 | M | YES | NO |
| CTRL | 33,48 | F | YES | NO |
| CTRL | 33,49 | F | YES | NO |
| CTRL | 33,75 | F | NO | YES |
| CTRL | 33,99 | M | YES | NO |
| CTRL | 34,94 | M | YES | NO |
| CTRL | 35,05 | F | YES | NO |
| CTRL | 35,67 | M | YES | NO |
| CTRL | 36,61 | F | YES | NO |
| CTRL | 36,93 | M | YES | NO |
| CTRL | 37,69 | M | YES | NO |
| CTRL | 37,80 | F | YES | YES |
| CTRL | 39,00 | F | YES | NO |
| CTRL | 39,51 | M | YES | NO |
| CTRL | 39,78 | F | YES | NO |
| CTRL | 39,81 | F | YES | NO |
|  |  |  |  |  |
| CTRL | 40,13 | F | YES | NO |
| CTRL | 40,50 | F | YES | NO |
| CTRL | 40,93 | F | YES | YES |
| CTRL | 41,89 | F | YES | NO |
| CTRL | 42,32 | F | YES | YES |
| CTRL | 42,58 | F | YES | NO |
| CTRL | 43,08 | F | YES | NO |
| CTRL | 43,75 | M | YES | NO |
| CTRL | 43,99 | F | YES | NO |
| CTRL | 44,39 | F | YES | NO |
| CTRL | 44,56 | M | YES | NO |
| CTRL | 44,58 | F | YES | NO |
| CTRL | 44,62 | F | YES | YES |
| CTRL | 44,66 | F | YES | YES |
| CTRL | 45,26 | M | YES | NO |
| CTRL | 45,32 | F | YES | YES |
| CTRL | 45,54 | M | YES | NO |
| CTRL | 46,43 | M | YES | NO |
| CTRL | 46,48 | F | YES | NO |
| CTRL | 46,86 | M | YES | YES |
| CTRL | 47,27 | F | YES | NO |
| CTRL | 47,61 | F | YES | NO |
| CTRL | 48,02 | M | YES | NO |
| CTRL | 48,20 | F | YES | NO |
| CTRL | 48,34 | F | YES | NO |
| CTRL | 49,03 | M | YES | YES |
| CTRL | 49,49 | F | YES | NO |
| CTRL | 49,79 | F | YES | YES |
| CTRL | 49,82 | F | YES | YES |
| CTRL | 50,23 | M | YES | YES |
| CTRL | 50,33 | M | YES | YES |
| CTRL | 50,79 | M | YES | NO |
| CTRL | 51,26 | M | YES | YES |
| CTRL | 51,33 | M | YES | NO |
| CTRL | 51,59 | F | YES | NO |
| CTRL | 51,65 | F | YES | NO |
| CTRL | 51,87 | M | YES | YES |
| CTRL | 51,88 | M | YES | YES |
| CTRL | 51,98 | F | YES | NO |
| CTRL | 52,46 | M | YES | NO |
| CTRL | 52,48 | F | YES | NO |
| CTRL | 52,91 | M | YES | YES |
| CTRL | 53,41 | M | YES | YES |
| CTRL | 53,64 | F | YES | YES |
| CTRL | 53,75 | F | YES | NO |
| CTRL | 53,80 | M | YES | NO |
| CTRL | 54,19 | M | YES | NO |
| CTRL | 54,58 | M | YES | NO |
| CTRL | 54,58 | F | YES | NO |
| CTRL | 54,72 | M | YES | YES |
| CTRL | 55,26 | F | YES | NO |
| CTRL | 55,84 | M | YES | NO |
| CTRL | 56,87 | F | YES | NO |
| CTRL | 57,14 | M | YES | NO |
| CTRL | 57,34 | F | YES | YES |
| CTRL | 57,57 | M | YES | NO |
| CTRL | 58,16 | F | YES | YES |
| CTRL | 58,31 | F | YES | NO |
| CTRL | 58,74 | F | YES | YES |
| CTRL | 59,73 | F | YES | NO |
| CTRL | 59,98 | M | YES | YES |
|  |  |  |  |  |
| CTRL | 60,19 | M | YES | YES |
| CTRL | 60,80 | F | YES | YES |
| CTRL | 61,41 | M | YES | NO |
| CTRL | 61,45 | F | YES | YES |
| CTRL | 62,50 | M | YES | YES |
| CTRL | 63,24 | M | YES | YES |
| CTRL | 63,81 | F | YES | YES |
| CTRL | 64,20 | M | YES | YES |
| CTRL | 64,73 | M | YES | YES |
| CTRL | 64,89 | M | YES | YES |
| CTRL | 65,16 | F | YES | YES |
| CTRL | 65,69 | F | YES | YES |
| CTRL | 66,07 | M | YES | YES |
| CTRL | 66,24 | M | YES | NO |
| CTRL | 66,65 | M | YES | YES |
| CTRL | 66,65 | M | YES | NO |
| CTRL | 67,45 | M | YES | NO |
| CTRL | 67,94 | M | YES | YES |
| CTRL | 67,95 | F | YES | NO |
| CTRL | 68,11 | M | YES | NO |
| CTRL | 68,16 | F | YES | NO |
| CTRL | 68,26 | F | YES | NO |
| CTRL | 68,44 | F | YES | NO |
| CTRL | 68,45 | F | YES | YES |
| CTRL | 69,40 | F | YES | NO |
| CTRL | 69,89 | F | YES | NO |
| CTRL | 70,33 | F | YES | NO |
| CTRL | 70,55 | M | YES | NO |
| CTRL | 71,53 | F | YES | NO |
| CTRL | 72,15 | M | YES | NO |
| CTRL | 72,21 | F | YES | NO |
| CTRL | 72,46 | F | YES | NO |
| CTRL | 72,48 | F | YES | NO |
| CTRL | 72,82 | F | YES | NO |
| CTRL | 73,09 | M | YES | NO |
| CTRL | 73,90 | F | YES | NO |
| CTRL | 74,06 | F | YES | NO |
| CTRL | 74,68 | M | YES | NO |
| CTRL | 74,74 | M | YES | NO |
| CTRL | 75,70 | M | YES | NO |
| CTRL | 76,27 | M | YES | NO |
| CTRL | 76,33 | M | YES | NO |
| CTRL | 76,95 | M | YES | NO |
| CTRL | 78,06 | F | YES | NO |
| CTRL | 78,06 | M | YES | YES |
| CTRL | 78,37 | F | YES | YES |
| CTRL | 78,84 | F | YES | YES |
| CTRL | 79,75 | F | YES | NO |
| CTRL | 79,92 | F | YES | NO |
|  |  |  |  |  |
| CTRL | 81,03 | F | YES | YES |
| CTRL | 81,10 | F | YES | YES |
| CTRL | 81,27 | F | YES | YES |
| CTRL | 82,41 | M | YES | NO |
| CTRL | 82,90 | M | YES | NO |
| CTRL | 84,49 | M | YES | NO |
| CTRL | 86,93 | F | YES | YES |
| CTRL | 87,43 | M | YES | YES |
| CTRL | 89,47 | M | YES | YES |
| CTRL | 89,88 | F | YES | YES |
| CTRL | 89,88 | M | YES | NO |
| CTRL | 94,01 | M | YES | YES |
| CTRL | 96,58 | F | YES | YES |
| CTRL | 97,35 | F | YES | YES |
|  |  |  |  |  |
|  |  |  |  |  |
| TBD24-*TERT* | 18,63 | F | YES | YES |
| TBD23-*TERT* | 22,03 | F | YES | YES |
| TBD27-*TERT* | 29,34 | M | YES | YES |
| TBD33-*RTEL1* | 40,2 | F | YES | YES |
| TBD32-*TERT* | 43,8 | M | YES | YES |
| TBD25-*TERT* | 48,54 | F | YES | YES |
| TBD3-*TERT* | 51,70 | M | YES | YES |
| TBD21-*TERC* | 56,2 | M | YES | YES |
| TBD26*-RTEL1* | 58,02 | F | YES | YES |
| TBD1-*TERT* | 60,81 | F | YES | NO |
| TBD22-*RTEL1* | 62,15 | F | YES | YES |
