# Supplementary material for: Telomere Dysfunction and Proteostasis Decline Define Distinct Pathways of Cellular Senescence in the Human Respiratory Tract
Source: Aging Cell. 2026 Apr 20;25(5):e70512. doi: 10.1111/acel.70512 (PMC13096579; doi:10.1111/acel.70512)
Supplement: Supplementary file 7 — Table S3: Antibodies used in this study. All antibodies were used in IF experiments. [file ACEL-25-e70512-s008.docx]

**Supplementary Table 3. Antibodies used in this study.** All antibodies were used in IF experiments.

| **Target** | **Species** | **Reference** |  |
| --- | --- | --- | --- |
| β-catenin | Rabbit | 8480S, Cell Signaling Technology |  |
| CD68 | Mouse | NB100-683B, Novus Biologicals |  |
| LAMP2 | Mouse | 66301-1-Ig, ProteinTech |  |
| Phospho-H2A.X (Ser139) (γ-H2AX) | Mouse | 05-636, Millipore |  |
| TRF2 | Rabbit | NB110-57130, Novus Biologicals |  |
| Prosurfactant Protein C | Rabbit | AB3786, Millipore |  |
| Mouse IgG (Alexa Fluor 647) | Donkey | A-31571, Invitrogen |  |
| Rabbit IgG (Alexa Fluor 488) | Goat | A-11008, Invitrogen |  |
|  |  |  |  |
