# Supplementary material for: Telomere Dysfunction and Proteostasis Decline Define Distinct Pathways of Cellular Senescence in the Human Respiratory Tract
Source: Aging Cell. 2026 Apr 20;25(5):e70512. doi: 10.1111/acel.70512 (PMC13096579; doi:10.1111/acel.70512)
Supplement: Supplementary file 8 — Table S4: Primers used in this study for qRT‐PCR. All primers were obtained from Eurogentec (Belgium). [file ACEL-25-e70512-s003.docx]

**Supplementary Table 4. Primers used in this study for qRT-PCR.** All primers were obtained from Eurogentec (Belgium).

| **Target** | **Forward primer** | **Reverse primer** |
| --- | --- | --- |
| ***ACTB*** | 5’-*GCTGGAAGGTGGACAGCGA* | 5’- *TGTACGCCAACACAGTGCTG* |
| ***CD68*** | *5’-TCCAGGGAAGCTGTGAGGGT* | *5’ -AGCCGAGAATGTCCACTGTGC* |
| ***p16*** | 5’-*GGAAGGTCCCTCAGACATCC* | 5’-*TACGAAAGCGGGGTGGGTTG* |
| ***p21*** | 5’-*GCAGACCAGCATGACAGATTT* | 5’-*GGATTAGGGCTTCCTCTTGGA* |
